# Longitudinal analysis of high-risk HPV infections reveals within-host viral genome changes over time

**DOI:** 10.1371/journal.ppat.1014362

**Published:** 2026-07-15

**Authors:** Sambit K. Mishra, Meredith Yeager, Chase W. Nelson, Bin Zhu, Laurie Burdett, Robert D. Burk, Michael Dean, Rolando Herrero, Mark Schiffman, Ana Cecilia Rodriguez, Lisa Mirabello

**Affiliations:** 1 Division of Cancer Epidemiology and Genetics, National Cancer Institute, National Institutes of Health, Rockville, Maryland, United States of America; 2 Cancer Genomics Research Laboratory, Frederick National Laboratory for Cancer Research, Rockville, Maryland, United States of America; 3 Department of Biology, Hood College, Frederick, Maryland, United States of America; 4 Department of Pediatrics, Epidemiology and Population Health, Microbiology and Immunology, and Obstetrics and Gynecology and Women’s Health, Albert Einstein College of Medicine, Bronx, New York, United States of America; 5 Agencia Costarricense de Investigaciones Biomédicas (ACIB-FUNIN), Formerly Proyecto Epidemiológico Guanacaste, Fundación INCIENSA, San José, Costa Rica; University of North Carolina at Chapel Hill, UNITED STATES OF AMERICA

## Abstract

Persistent infection with high-risk (HR)-HPV causes cervical cancer, however, it is unclear why most infections resolve while a minority progress. We deep sequenced the HPV genomes of 1,228 HR-HPV-positive serial samples from 351 women with persistent infections (2–10 serial samples per woman over 1–8 years), including 279 controls and 72 precancer/cancer cases, to assess HR-HPV genome changes during infection and relation to infection outcomes. Seventy-seven percent of persistent infections (45–97% by HPV type) were infections with the same exact viral genome isolate; for HPV16, only 52% were persistent with the same isolate. This may suggest some infections include a type-specific isolate switch or new isolate infection during persistence. We additionally observed within-host change to the HPV genome estimated as gradual changes to intrahost single nucleotide variant (iSNV) frequency, and changes varied by HPV type, with HPV33 infections showing the most iSNV changes. Cases exhibited fewer viral genome changes during infection compared to controls (OR = 0.31, 95% CI = 0.1 – 0.86, p = 0.019), suggesting a more stable and clonal viral genome in cases. By viral gene, E7 had fewer nonsynonymous mutations in the cases compared to controls that cleared within 2 years of infection (p = 0.012), which confirms the importance of E7 conservation and suggests mutations to E7 reduce persistence associated with progression. There was a similar pattern in E4 (p = 0.013), while E5 had more changes in the cases (p = 0.008). A subset of 28 infections had an intervening HPV-negative sample between HPV-positive visits; 93% of these infections had the same exact viral genome isolate in the samples before and after the negative, consistent with subclinical persistence and subsequent re-detection. Our data suggests that HR-HPV type-persistence can include a collection of viral isolates, and viral mutations during infection, particularly in E7, reduce HR-HPV persistence and thus carcinogenic potential.

## Author summary

Cervical cancer is the fourth most common cancer in women worldwide and is caused by persistent infection with high-risk types of human papillomavirus. Although most infections are cleared naturally, a small number persist and can progress to precancer and cancer. It remains unclear why certain infections progress while others do not. In this study, we followed women over time and analyzed repeated samples from the same infections to examine how the viral genome changes within an individual during infection. We found that the infections that subsequently progressed to precancer or cancer had significantly more stable viral genomes, whereas infections that did not progress showed greater genetic variation over time. We also observed differences in specific viral genes between persistence associated with progression and infections that cleared. These findings suggest that changes within the viral genome during infection can influence disease outcome. By highlighting the importance of tracking viral genetic variation over time, our work provides new insight into HPV infections and suggests that monitoring viral genome stability could help improve risk assessment and prevention strategies.

## Background

Each year, high-risk human papillomaviruses (HR-HPVs) cause approximately 690,000 cancers worldwide, including 660,000 cervical cancers and 348,000 deaths due to cervical cancer [1]. Persistent infection with one of the 12 HR-HPV types (HPV16, 18, 31, 33, 35, 39, 45, 51, 52, 56, 58, 59) is responsible for virtually all cases of cervical cancer [2–4]. HPVs are classified based on their ~7.9 kb double-stranded DNA genome, and the HR-HPVs are all clustered in the *Alphapapillomavirus* genus in four species: Alpha-9 (HPV16, 31, 33, 35, 52, 58), Alpha-7 (HPV18, 39, 45, 59), Alpha-5 (HPV51), and Alpha-6 (HPV56) [5]. The majority of HR-HPV infections are cleared by the immune system; only a small fraction will persist and progress to precancer and subsequently cancer [6,7]. Carcinogenicity varies by HR-HPV type. HPV16 is the most common carcinogenic type (responsible for ~60% of cervical cancers), followed by HPV18 (responsible for ~10–15%), while the other HR-HPV types including those genetically closely related to HPV16 and HPV18 (i.e., HPV31/HPV35 and HPV45, respectively) are much less carcinogenic (each responsible for ≤6% of cervical cancers) [8]. Within each type, genetic variation has been further linked to differences in HPV carcinogenicity by lineages and sublineages, and even individual single nucleotide polymorphisms (SNPs) [9–12].

The HPV genome is considered evolutionarily stable, however, there have been only a few studies evaluating within-host HPV evolution, and recent studies suggest that the virus genome can change during an infection. Large studies investigating within-type genome diversity have shown a high level of viral isolate diversity at the consensus sequence level (i.e., a single representative sequence with the most common nucleotide at each position) among women in a given population [11,13,14], as well as intrahost single nucleotide variants (iSNV) within women [14–18]. Host innate antiviral APOBEC3 cytidine deaminases can induce HPV genome mutations [19] and contribute to HPV diversity, in addition to inducing human somatic driver mutations [20]. We have shown that HPV16 mutations potentially induced by APOBEC3 frequently occur at low variant allele fractions (VAF) and that infections with more APOBEC3 mutations are more likely to be transient [15].

It is unclear how often the viral genome changes within a woman during her infection and if these changes impact infection outcome. Although most HPV genetic studies have been cross-sectional, a few longitudinal studies have evaluated HPV16 genome variation during infection. One study documented HPV16 genome changes in 94 women that later developed high-grade lesions using two serial samples (0.5–178 months apart) and reported that the majority had no genome changes during persistent infection, while 44% had 1–15 nucleotide changes [21]. Another study of 115 women with 1–4 serial samples over three years identified a large amount of diversity among the women and found that the HPV16 genomes were conserved among serial samples within the women during infection [13]. Of note, both of these studies, and many of the earlier cross-sectional studies, focused on changes to the consensus-level viral genome sequence (i.e., the most common nucleotide detected in the sequence reads at each site). While this approach is well suited for sublineage assignment and population-level comparisons across women, it excludes changes occurring in a minority of reads and therefore likely overlooks many viral genomic changes within a host. In contrast, a recent longitudinal study evaluated HPV16 iSNV changes over time in 40 women (2–5 serial samples per woman) and reported fewer iSNVs in the high-grade lesions compared to the normal/low-grade lesions, and an increase in APOBEC3-induced iSNVs over time in the normal/low-grade lesions [16].

To our knowledge, no large studies have evaluated HR-HPV isolate persistence or temporal changes to the allelic proportions of iSNVs over the duration of infection across HR-HPV types and the risks associated with such changes. In this study, we investigated HR-HPV isolate persistence and longitudinal iSNV dynamics using whole-genome sequencing data from women enrolled in the prospective Guanacaste, Costa Rica Natural History Study and assessed their associations with infection outcome. We analyzed 1,228 serial samples (2–10 serial samples per woman) from 351 women positive for one or more of the 12 HR-HPV types. To capture within-host viral genomic changes across the full spectrum of variant allele fractions (VAFs), we performed whole-genome sequencing of all serial samples for each HR-HPV type. We investigated viral isolate (i.e., genome sequence) persistence, and iSNV VAF changes over time and how they relate to infection clearance or precancer/cancer progression. In addition, we evaluated the HR-HPV genomes in the women with type-specific HR-HPV persistent infections and an intervening negative between HR-HPV positive samples to assess genomic evidence for a subclinical infection (i.e., lack of detection) of the same viral isolate.

## Results

**Fig 1:**
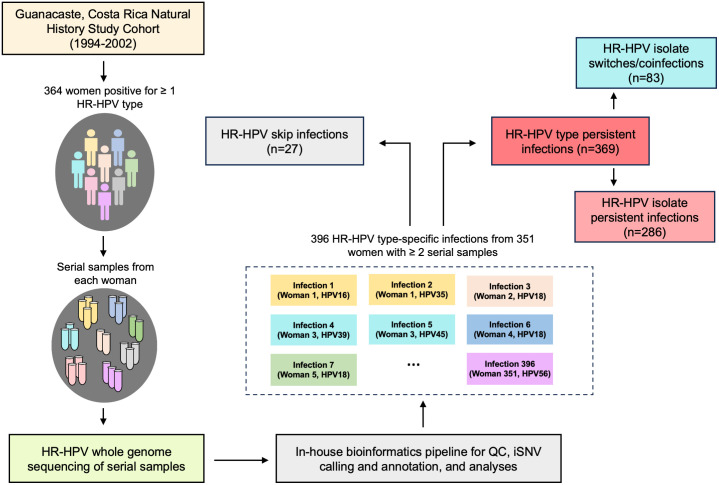
Overview of the study workflow and methodology. 364 women (depicted as unique individuals by the different colors) positive for ≥1 HR-HPV types were selected from the Guanacaste, Costa Rica Natural History Study. Each woman had multiple serial screening visits during the study and was classified as a case if she progressed to cervical intraepithelial neoplasia grade 2 or higher (CIN2+) or as a control if she cleared/controlled her infection and never progressed to CIN2+. Exfoliated cervical serial samples (shown as tubes, colored to match the woman) were collected from multiple screening visits; the number of serial samples collected per woman ranged from 2 to 10. Each HR-HPV+ sample was HPV whole-genome sequenced. Each HR-HPV type detected in a woman (HR-HPV type-specific infection) was evaluated separately. The infections were categorized in one of two groups: skip infections (n = 27) with intervening HPV-negative tests or HR-HPV type persistent infections without any intervening HPV-negative tests (n = 369). The type-persistent infections were then evaluated for isolate persistence (i.e., whether each serial sample of the infections had the same HPV consensus genome sequence or viral isolate in each serial sample) and iSNV frequency changes (i.e., changes to the proportion of viral sequence reads within the host having a particular single nucleotide variant) during the infection.

We evaluated 396 HR-HPV type-specific infections from 351 women with ≥ 2 serial samples (1,228 total samples) and HPV genome sequence data that passed our quality control (Fig 1). The characteristics of the 351 women are shown in Table 1, and by the 12 HR-HPV types in S1 Table. In total, we included 279 controls with benign or low-grade infections (≤CIN1; cervical intraepithelial neoplasia grade 1 or lower), and 72 cases including 28 CIN2, 40 CIN3 and 4 cancers. At study enrollment, 40% of the women had a co-infection with more than one HR-HPV type and 61% were less than 40 years of age (Table 1). HPV16 was the most common HR-HPV (23.7% of infections), followed by HPV31 (13.6%) and HPV58 (11.4%). Grouping the HR-HPV types by species, 267/396 (67.4%) of the infections were Alpha-9 HPV types, 69/396 (17.4%) were Alpha-7 types, and 60/396 (15.2%) were Alpha-5/Alpha-6 types.

**Table 1:** Characteristics and HR-HPV types detected in 351 women in the Guanacaste, Costa Rica Natural History Study.

Characteristics	N (%)
**Enrollment Age**	
18-29	135 (38.5)
30-39	79 (22.5)
40-49	46 (13.1)
50-59	33 (9.4)
60+	58 (16.5)
**Histology**	
≤ CIN1	279 (79.5)
CIN2	28 (8)
CIN3	40 (11.4)
Cancer	4 (1.1)
**HR-HPV type co-infection**	
Yes	141 (40)
No	210 (60)
Total	351 (100%)
**HR-HPV type**	
16	94 (23.7)
18	31 (7.8)
31	54 (13.6)
33	24 (6.1)
35	16 (4)
39	16 (4)
45	16 (4)
51	22 (5.6)
52	34 (8.6)
56	38 (9.6)
58	45 (11.4)
59	6 (1.5)
Total*	396 (100%)

*Total number of HR-HPV infections, women coinfected with ≥ 1 type are counted more than once.

### HR-HPV type-specific persistence can represent persistence of multiple viral isolates

**Fig 2:**
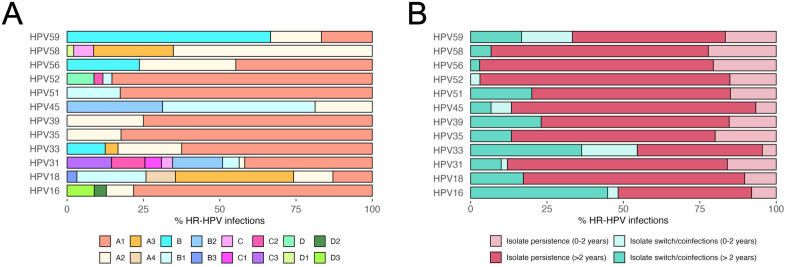
Distribution of sublineages (A) and frequency of viral persistence at the isolate-level (B) for each HR-HPV type. **(A)** The percentage of each sublineage observed per HR-HPV type. **(B)** Distribution of isolate persistence (i.e., same consensus HPV genome sequence observed in all serial samples; n = 286) and isolate switches (i.e., different consensus HPV genome sequence with ≥2 nucleotide changes observed across serial samples; n = 83) stratified by infection duration (0-2 years or >2 years) for each HR-HPV type across all type-persistent infections.

For each of the 396 HR-HPV type-specific persistent infections, a sublineage was assigned to each serial sample (Fig 2A). Lineage A was most common across nine HR-HPV types: 87% of HPV16 infections; 74% of HPV18; 88% of HPV33; 100% of HPV35; 100% of HPV39; 83% of HPV51; 85% of HPV52; 76% of HPV56; and 91% of HPV58. For HPV16, sublineage A1 was most common (78%), followed by D3 and A2 (each 9%), and D2 (4%). At least two sublineages were detected for each HR-HPV type, and HPV31 had the greatest number of sublineages (n = 8). Twenty-seven (7%) of the 396 infections were classified as skip infections with ≥1 intervening HPV-negative samples and were analyzed separately. The remaining 369 persistent infections (n = 1,193 serial samples) were further categorized as ‘isolate persistence’ or ‘isolate switch/coinfection’, depending on whether the same viral genome isolate, or a different viral genome isolate (i.e., genome with ≥2 nucleotide changes) was observed among the serial samples (see methods for more details).

Isolate persistence was variable by HPV type and the majority of the infections across the HR-HPV types persisted for more than two years (83%, n = 307) (Fig 2B, S2 Table). Notably, for HPV16 and HPV33, 48% (42/87) and 55% (12/22) of the type-level persistent infections were not persistent at the viral isolate-level; that is, the same viral genome sequence was not detected throughout the infection period. Instead, isolate switches were observed, suggesting a new infection with the same type. Overall, 77.5% (286/369) of all the HR-HPV type persistent infections were persistent at the isolate-level with the same consensus viral genome throughout the infection period, whereas isolate switches/coinfections were observed in 22.5%. Interestingly, HPV33 infections persisting for 0–2 years had the highest frequency of isolate switches (80%), and HPV16 had the highest percentage of switches (51%) in infections that persisted for more than two years (S2 Table).

Among the serial samples (n = 1,193) of a given woman’s type-specific persistent infection, the majority of isolate sequences were exactly the same (79%; S1 Fig). In contrast, type-specific exhaustive pairwise comparisons of viral isolates between all the women positive for that type, comparing only the enrollment sample viral isolates, showed that there was a high level of isolate diversity among women in the population (isolates differed by two or more nucleotides), ranging from 81% to 100% (S2 Fig). The level of isolate diversity detected per type did not significantly correlate with the percentage of switches/coinfections (Pearson correlation, r = 0.27).

### Evidence for within-host iSNV changes during HR-HPV type-specific persistent infections

**Fig 3:**
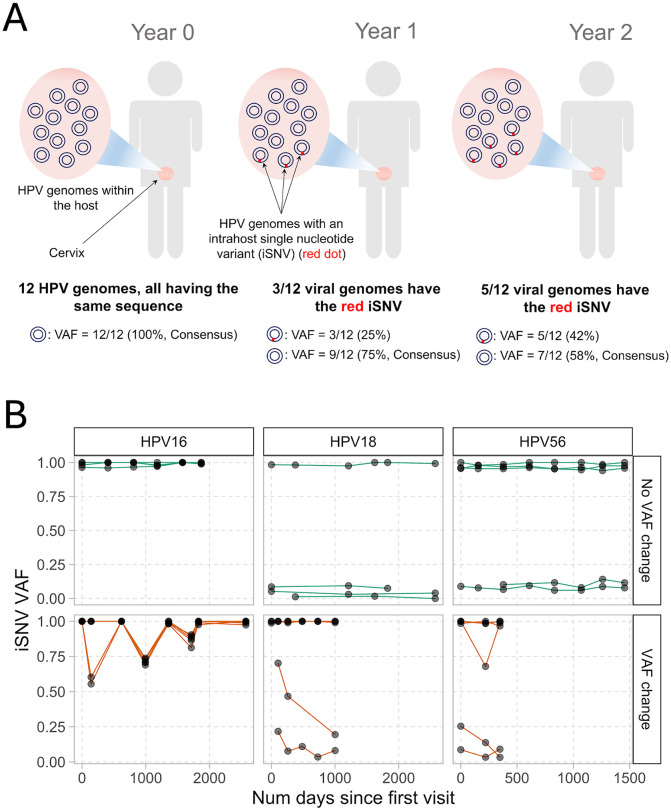
Within-host iSNV VAF changes. **(A)** A cartoon explaining the evolution of the viral genome within a woman at the sub-consensus level. At year 0, the woman has 12 copies of the viral genome, all with the same sequence. Within a year, 3 of 12 viral genomes have accumulated the red iSNV. After two years of infection, the proportion of genomes (VAF) with the red iSNV has increased from 0 → 25% → 42%. However, because the red iSNV is still present in a minority of viral genomes (<50%), the consensus sequence remains unchanged. **(B)** Examples of our HPV16, HPV18 and HPV56 infections without any significant iSNV VAF changes (top) and with at least one significant iSNV VAF change (bottom). Each iSNV is shown as a distinct line and the observed VAF at each serial time point as a dot. iSNV, intrahost single nucleotide variant; VAF, variant allele fraction.

We evaluated 1,193 serial samples (excluding skip infections) from 369 HR-HPV type-specific persistent infections to identify iSNVs that significantly changed VAF during infection. Fig 3A illustrates a hypothetical scenario where within-host ΔiSNV_VAF_ (i.e., iSNV VAF changes) may not be detected in consensus-level sequence analyses; while the consensus viral sequence for this infection remains the same after two years, the proportion of viral genomes with the iSNV of interest increases from 0% to 25% to 42% within the host. Therefore, we evaluated iSNV changes across the full spectrum of variant frequencies, including both consensus and sub-consensus levels.

After stringent quality control filtering, we included 323 of 369 type-persistent infections with at least one iSNV observed in ≥ 2 serial samples in this analysis. Cumulatively, these infections harbored 1,957 iSNVs in ≥ 2 serial samples with a sequencing depth ≥ 100 and VAF ≥ 0.005 and ≤ 0.995 in at least one serial sample (our criteria for identifying iSNV/variable sites). Each of the 1,957 iSNVs were further categorized based on their VAF change, as either increasing, decreasing, or no change (see Methods for more details).

We identified a total of 389 iSNVs (19.9% of iSNVs) that significantly changed VAF over time. The distribution of iSNVs that significantly increased or decreased VAF by HPV type is shown in Table 2. Interestingly, the proportion of iSNVs with VAF increase/decrease varied across types. Of note, the three HR-HPV types considered the most carcinogenic (HPV16, HPV18, HPV45) [22] had similar levels of iSNV changes (~19%), as well as HPV35 (19.6%). HPV33 had the highest fraction of iSNVs with VAF changes (32.2%), while HPV56 had the least (7.1%). In addition, HPV51 had ≥ 3x iSNVs with VAF increase than decrease. We also show representative examples of infections with stable and changing iSNV VAFs during persistent infection (Fig 3B).

**Table 2:** Distribution of iSNVs by HPV type and iSNV VAF change.

HPV Type	Total iSNVs N	iSNVs with significant VAF changes		
		VAF increase N (%)	VAF decrease N (%)	Total N (%)
**HPV16**	177	15 (8.5%)	19 (10.7%)	34 (19.2%)
**HPV18**	131	8 (6.1%)	18 (13.7%)	26 (19.8%)
**HPV45**	122	7 (5.7%)	16 (13.1%)	23 (18.9%)
**HPV33**	261	48 (18.4%)	36 (13.8%)	84 (32.2%)
**HPV58**	327	46 (14.1%)	43 (13.1%)	89 (27.2%)
**HPV31**	191	9 (4.7%)	9 (4.7%)	18 (9.4%)
**HPV52**	172	10 (5.8%)	11 (6.4%)	21 (12.2%)
**HPV35**	138	13 (9.4%)	14 (10.1%)	27 (19.6%)
**HPV59**	26	3 (11.5%)	5 (19.2%)	8 (30.8%)
**HPV39**	118	20 (16.9%)	11 (9.3%)	31 (26.3%)
**HPV56**	240	7 (2.9%)	10 (4.2%)	17 (7.1%)
**HPV51**	54	8 (14.8%)	3 (5.6%)	11 (20.4%)

N, number of iSNVs with the given change (% of total iSNVs for that type); VAF, variant allele fraction. Order of types is based on the worldwide attribution to cancer.

Since the PCR amplification process is stochastic, it is possible that some amplicons may be amplified more than others, and therefore, some variant positions may have higher sequencing depth than others. To exclude the possibility that the observed iSNV VAF changes were due to sequencing depth differences across serial samples, we examined the correlation between standard deviation of variant depth and standard deviation of VAF and observed no correlation (r = 0.011) between the two variables (S3A Fig). We also observed a minimal correlation (r = 0.056) between VAF and sequencing depth for each variant position (S3B Fig), confirming that the observed variations in iSNV VAF were not linked to sequencing depth changes.

### Within-host viral iSNV frequency changes are associated with disease risk

**Fig 4:**
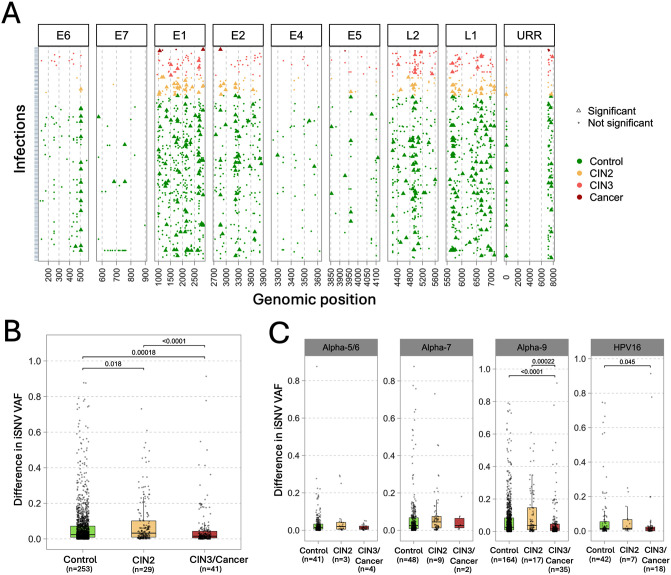
iSNV VAF changes across 323 HR-HPV type persistent infections by viral gene and infection outcome. **(A)** Each iSNV detected in the persistent HR-HPV type-specific infections is shown by HPV genome position, viral gene/region, and colored by infection outcome (Control, CIN2, CIN3, Cancer). The iSNVs with significant VAF changes are shown as triangles and those without significant changes as dots. Infections with at least one iSNV in any of the 9 genes shown above were considered. **(B)** Distribution of changes in iSNV VAF measured as the absolute of the difference between VAF at the first serial sample observation and last observation for each iSNV by infection outcome for all HR-HPV types and for **(C)** Alpha-5/6, Alpha-7 and Alpha-9 species groups. Distributions were compared using Wilcoxon’s test. Only significant p values (p  <  0.05) are shown. Numbers correspond to the number of infections in each outcome group for each clade. iSNV, intrahost single nucleotide variant; VAF, variant allele fraction. E6, early gene 6; E7, early gene 7; E1, early gene 1; E2, early gene 2; E4, early gene 4; E5, early gene 5; L2, late gene 2; L1, late gene 1; URR, upstream regulatory region. CIN2, cervical intraepithelial neoplasia grade 2; CIN3, cervical intraepithelial neoplasia grade 3; Cancer includes adenocarcinoma and squamous cell carcinoma.

The frequency of iSNV VAF changes in the controls was higher than in the precancer/cancer cases (CIN2, CIN3, Cancer) across all HR-HPV type persistent infections, particularly in the viral oncogenes E6, E7 and E5 (Fig 4A). Interestingly, several iSNV positions that exhibited significant frequency changes in controls were not variable in cases, with no iSNVs observed at these sites. As an orthogonal approach, we also estimated iSNV VAF changes by calculating the absolute of the difference between the iSNV VAF at first detection and at last detection across all HR-HPV types stratified by infection outcome (Fig 4B). There was a similar trend of lower iSNV VAF changes in CIN3/Cancer cases compared to controls (P = 0.0002). The distribution of VAF changes in women with CIN2 more closely resembled that of controls than CIN3/Cancer cases, which reflects their less severe and ambiguous histopathology. By HPV species (Fig 4C), we consistently observed the same trend across Alpha-9, Alpha-7 and Alpha-5/6 HPV types, although the comparisons were only significant for the Alpha-9 species likely due to the larger number of infections in this group. By HPV type, there was only enough controls/cases (n > 5) for this comparison for HPV16; less iSNV changes were also observed in the cases compared to controls for the HPV16 infections (Fig 4C).

We further classified each infection based on the presence of at least one iSNV with a significant frequency change, as either VAF-change or No-VAF-change and evaluated the associations with infection outcome (S4 Fig). Considering all HR-HPV types with ≥ 2 years persistence and ≥ 3 serial samples, we observed a significant association between no iSNV VAF changes and progression to precancer/cancer, suggesting a more stable HPV genome in cases than controls (CIN3 + : OR 0.15, 95% CI 0.03–0.63, P = 0.003; Table 3). This association remained after adjusting for enrollment age, number of sexual partners and HR-HPV co-infection status (CIN2 + : OR 0.38, 95% CI 0.14–0.98, P = 0.049). A similar trend was observed for the Alpha-9 type infections (Table 3). Case counts were too small for association testing for the Alpha-7 and Alpha-5/6 types (n < 5).

**Table 3:** HR-HPV iSNV VAF changes during a persistent infection and associations with infection outcome.

HPV Type	Persistence years	N visits	Infection outcome*	No-VAF-change	VAF-change	OR (95% CI)	P value
**All HR-HPV**	**≥ 2**	**≥ 3**	Control	34 (37.8%)	56 (62.2%)	Ref	
			CIN2+	16 (66.7%)	8 (33.3%)	0.31 (0.1,0.86)	**0.019**
			CIN3+	12 (80%)	3 (20%)	0.15 (0.03,0.63)	**0.003**
**Alpha-9**	**≥ 2**	**≥ 3**	Control	21 (35.6%)	38 (64.4%)	Ref	
			CIN2+	13 (61.9%)	8 (38.1%)	0.34 (0.11,1.07)	**0.043**
			CIN3+	10 (76.9%)	3 (23.1%)	0.17 (0.03,0.76)	**0.011**

* Case HR-HPV coinfections with sequencing data were excluded from this analysis.

Ref: referent group.

Odds ratios (OR), 95% confidence intervals (95% CI) and P values were calculated using a two-sided Fisher’s exact test.

No-VAF-change is the number of infections that were categorized as No-VAF-change; VAF-change is the number of infections that were categorized as VAF-change.

N visits is the number of serial screening visits in which an infection was detected.

Persistence years was estimated as the time between the first positive serial sample (i.e., screening visit) collection date and the last positive serial sample collection date for each HR-HPV type, divided by 365.

### Gene-specific nonsynonymous viral mutations are associated with infection outcome

To determine if nonsynonymous single nucleotide changes in specific genes were associated with viral clearance, we categorized the HR-HPV type-persistent infections based on HR-HPV clearance [‘control/clearance’ (n = 153) and ‘control/no clearance’ (n = 132)] and on the duration of infection prior to clearance (S5, S6 Figs). Based on the infection clearance time, we further stratified the control/clearance infections into short-term and long-term clearance groups, ‘clearance (0–2 years)’ and ‘clearance (4+ years)’. We evaluated all single nucleotide variants with VAF ≥ 0.005, sequencing depth ≥ 100 and appearing in at least two serial samples, excluding variant sites consistent with a sublineage coinfection of that HR-HPV type (see Methods for more details; S1 File). We compared the proportions of nonsynonymous single nucleotide variants by viral gene between the clearance (0–2 years), clearance (4 + years) and the cases (CIN2+).

**Fig 5:**
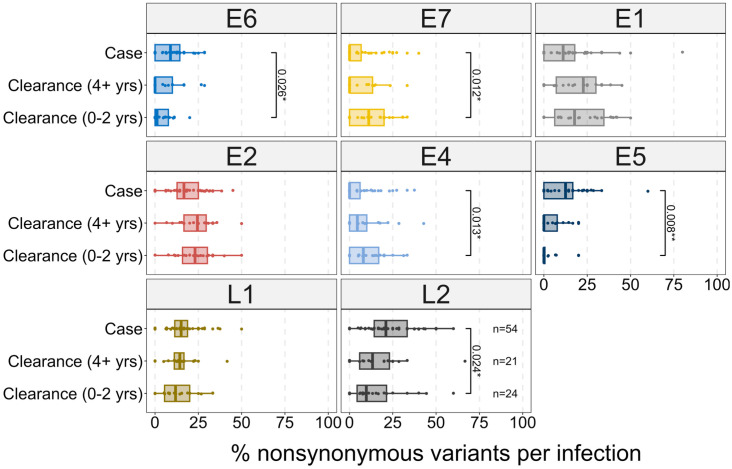
Distribution of nonsynonymous single nucleotide variants by gene and infection outcome. The percentage of nonsynonymous changes (iSNVs and SNVs) per infection per gene is shown for CIN2+ cases, Controls/Clearance (4+ years) and Controls/Clearance (0–2 years). Only infections with at least 3 nonsynonymous changes were considered. P values were corrected with FDR correction and only significant P values (<0.05) are shown. The L2 gene panel shows the number of infections for each outcome category. CIN2+, cervical intraepithelial neoplasia grade 2 or higher; ORF, open reading frame, E6, early gene 6; E7, early gene 7; E1, early gene 1; E2, early gene 2; E4, early gene 4; E5, early gene 5; L2, late gene 2; L1, late gene 1; * (p < 0.05), ** (p < 0.01).

Among infections with nonsynonymous single nucleotide variants, the cases had significantly fewer nonsynonymous variants in E7 and E4 ORFs, compared to the clearance (0–2 years) group (Fig 5). The most significant differences in nonsynonymous variant distributions were between the more rapid clearance (0–2 years) group and the cases. The clearance (4 + years) group was more similar to cases in E7, with a lower proportion of nonsynonymous mutations. Interestingly, we observed the opposite pattern for the E5, L2, and E6 ORFs: the cases had significantly more nonsynonymous variants compared to the clearance (0–2 years) group (Fig 5). Similar patterns were observed when considering only the Alpha-9 HPV types (S7A Fig) and only the A1/A2 sublineage infections across all the HR-HPV types (S7B Fig). We did not observe any significant patterns for the Alpha-7 types likely due to the small numbers.

Categorizing infections based on number of nonsynonymous single nucleotide variants (none vs. ≥ 1), there was a strong inverse association between nonsynonymous iSNVs in E7 and E4 and infection outcome comparing CIN2 + cases to the rapid clearance (0–2 years) controls (E7: OR 0.19, 95% CI 0.06–0.6, P = 0.012; E4: OR 0.23, 95% CI 0.07–0.71, P = 0.016) (Table 4), suggesting that infections that cleared within two years had more nonsynonymous mutations in E7 and E4 compared to the cases. In contrast, nonsynonymous changes in the E5 gene were detected less in the controls, suggesting that the cases had more nonsynonymous mutations in E5 (OR 4.99, 95% CI 1.57–18.03, P = 0.012) (Table 4). No other associations were significant. The associations and distributions of nonsynonymous mutations by gene were also calculated separately for Alpha-9 types (S3 Table) and similar significant associations were observed.

**Table 4:** Nonsynonymous single nucleotide changes associated with infection outcome by viral gene for all HR-HPV infections.

Gene	Infection outcome*	No Nonsynonymous changes	≥ 1 Nonsynonymous changes	OR (95% CI)	P value^#^
**E7**	Clearance (0–2 yrs)	7 (29.2%)	17 (70.8%)	Ref	
	CIN2+	37 (68.5%)	17 (31.5%)	0.19 (0.06,0.6)	**0.012**
**E5**	Clearance (0–2 yrs)	18 (75%)	6 (25%)	Ref	
	CIN2+	20 (37%)	34 (63%)	4.99 (1.57,18.03)	**0.012**
**E4**	Clearance (0–2 yrs)	8 (33.3%)	16 (66.7%)	Ref	
	CIN2+	37 (68.5%)	17 (31.5%)	0.23 (0.07,0.71)	**0.016**
**L2**	Clearance (0–2 yrs)	6 (25%)	18 (75%)	Ref	
	CIN2+	4 (7.4%)	50 (92.6%)	4.08 (0.86,22.05)	0.122
**L1**	Clearance (0–2 yrs)	5 (20.8%)	19 (79.2%)	Ref	
	CIN2+	6 (11.1%)	48 (88.9%)	2.08 (0.45,9.33)	0.361
**E1**	Clearance (0–2 yrs)	6 (25%)	18 (75%)	Ref	
	CIN2+	21 (38.9%)	33 (61.1%)	0.53 (0.15,1.68)	0.361
**E6**	Clearance (0–2 yrs)	12 (50%)	12 (50%)	Ref	
	CIN2+	19 (35.2%)	35 (64.8%)	1.83 (0.62,5.46)	0.361
**E2**	Clearance (0–2 yrs)	2 (8.3%)	22 (91.7%)	Ref	
	CIN2+	7 (13%)	47 (87%)	0.61 (0.06,3.59)	0.713

Only infections with ≥ 3 nonsynonymous mutations were considered.

‘No Nonsynonymous changes is the number of infections with no nonsynonymous iSNVs/SNVs, ‘≥ 1 Nonsynonymous changes’ is the number of infections with at least 1 nonsynonymous iSNVs/SNVs.

Odds ratios (OR), 95% confidence intervals (95% CI) and P values were calculated using a two-sided Fisher’s exact test; to enable calculations for Fisher exact test, the counts were incremented by 0.6 if any count in a 2x2 contingency table was 0.

#FDR correction was applied to the P values; significant p values (<0.05) are shown in bold.

CIN2+ includes cervical intraepithelial neoplasia grade 2 or higher.

* CIN2+ cases compared to the rapid clearance (0–2 years) controls; Coinfected cases with sequencing data were excluded.

### Intervening negative ‘skip infections’: Evidence for lack of detection followed by re-appearance of the same virus

**Fig 6:**
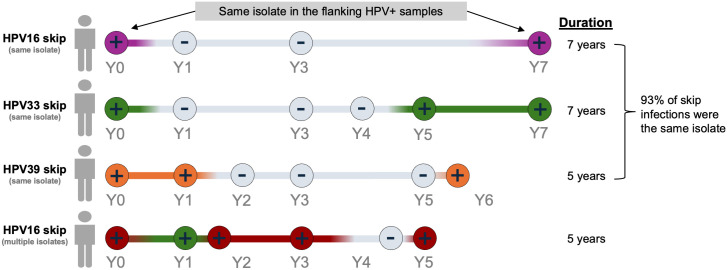
Illustration of skip infections with intervening negative tests and viral isolates detected. Of the 26 HR-HPV skip infections observed in our study, we illustrate the viral isolates detected for four. Each row is a separate skip infection, the visit years are denoted as Y0, Y1, Y2, etc., each serial sample is depicted as a circle, and the duration of the infection is reported for each. Serial samples negative for the HR-HPV type are colored grey and denoted with a ‘-’. Positive ‘+’ samples that had the same isolate are shown in the same color, while those with a different isolate are shown with a different color. The horizontal bars connecting the circles represent the serial timeline for each infection.

To evaluate if there was evidence of a subclinical persistent infection or lack of detection followed by re-appearance, we identified 27 HR-HPV type-specific infections that had an HPV-negative test (no detection) for a HR-HPV type within a string of HPV-positive tests (detection) for that type (i.e., HR-HPV negative with flanking HR-HPV positive tests; termed ‘skip infections’). One of the 27 identified skip infections was excluded from analysis due to insufficient sequence coverage across the viral genome in the flanking samples for thorough evaluation. Two of the 26 (7.7%) infections had two independent skip events (e.g., an infection with the following HPV test results: ++ - ++ - ++), therefore, a total of 28 skip events were observed in the 26 infections (Table 5). We determined the HR-HPV sublineage and then compared the entire viral genome sequence (isolate) at every nucleotide position for each serial sample in each skip infection series to determine if the same exact virus was present at all time points. 92.9% (26 of 28) of the skip infections had the same exact HPV genome isolate in the flanking serial samples, suggesting that the same HR-HPV was present throughout the infection (Table 5). There were 2 infections that had a different isolate after the tested negative sample, and one had entirely different sublineages in the flanking samples (HPV35 A1 vs. A2). We illustrate four of the skip infections from this set in Fig 6; three of these included infections had the exact same isolate observed in each of the positive serial samples and flanking samples over 5–7 years of persistence, while the fourth had 2 different isolates detected in the 5 positive serial samples.

**Table 5:** Summary of the 26 HR-HPV skip infections with an intervening HR-HPV negative test.

HPV Type	No. of skip infections	Same isolate in the flanking positive samples, N (%)
HPV16	6	5 (83%)
HPV18	2	2 (100%)
HPV31	4	4 (100%)
HPV33	2	2 (100%)
HPV35*	1 (*2)	1 (50%)
HPV39	3	3 (100%)
HPV45	1	1 (100%)
HPV51	2	2 (100%)
HPV52	1	1 (100%)
HPV56*	4 (*5)	5 (100%)
HPV58	0	0 (0%)
HPV59	0	0 (0%)

* There were 2 independent skip infections in the same woman for HPV35 and HPV56.

Eighteen ‘skip infections’ had one negative test (i.e., one intervening negative) and 10 had two or more negative tests among the persistent positive serial samples (S4 Table). The enrollment age of the women who had a skip in their infection was older (median age of 38 years) than the women without a skip (median age of 34 years), although the age difference was not significant (S8 Fig).

## Discussion

We conducted the largest study to date comprehensively evaluating HPV genome changes during persistent infection across all HR-HPV types and the relation to infection outcome. The study is based on deeply sequenced HPV whole-genomes from 396 HR-HPV infections and 1,228 prospectively collected serial samples. We report several fundamental observations using this unique dataset: (1) the same consensus viral isolate was often not observed throughout HR-HPV type persistent infections indicating an isolate switch (i.e., a new or different genome isolate of the same HR-HPV type indicated by two or more distinct nucleotide changes), and ~50% of both HPV16 and HPV33 persistent infections included a new virus isolate or switch. (2) The infections that progress to precancer/cancer have significantly fewer iSNV frequency changes (i.e., within-host SNV changes indicated by a significant frequency change among serial samples) compared to the infections that do not progress; (3) the controls that clear their infections rapidly (≤ 2 years) have significantly more nonsynonymous mutations in E7 and less in E5 genes compared to precancer/cancer cases; (4) nearly all (93%) persistent infections with intervening negatives, ‘skip infections’, have the same exact HPV genome isolate in the flanking serial samples, suggesting that the same HR-HPV is present throughout the infection and likely undetected in the intervening negative sample(s) potentially due to subclinical persistence and subsequent re-appearance.

The prevalence of viral persistence at the isolate-level in the HR-HPV type persistent serial samples, i.e., identical genome sequence over time, was variable by HR-HPV type, ranging from 45% (HPV33) to 97% (HPV56) of all persistent infections. After HPV33, HPV16 had the highest frequency of a different viral isolate (isolate switch) detected among the serial samples of a persistent infection: approximately half of the type-level persistent infections did not represent persistence of the same virus. The majority (54/100) of isolate switch sequences had four or more nucleotide changes, and twenty-two of these had more than ten changes, consistent with a different type-specific sublineage. A sudden switch to a viral consensus genome sequence with a large number of nucleotide changes likely suggests infection with a new viral isolate of that HPV type. This would suggest that some HPV16 infections may be progressing to precancer and cancer in even less time of persistence than previously thought.

It’s also possible that a co-infection with a different isolate of the same type at one time point switched to the dominant isolate or clone at the next time-point. The isolate switches that differ by only a small number of changes (e.g., two nucleotide changes) could also result from rapid selection of variants in the population, potentially driven by host enzymes such as APOBEC3. A previous HPV16 longitudinal study reported a median of 0 substitutions/site/year (CI 0–0.8 × 10^−4^) in the consensus genome sequence, suggesting that the viral consensus-level sequences within the host were largely stable [23]. Therefore, we don’t anticipate rapid selection to account for many of the observed consensus changes. Across all HR-HPV types, we observed isolate-level persistence throughout 78% of infections, which is also consistent with the study by Arroyo-Mühr et al [21]. Notably, we and others have previously reported extensive HPV16 isolate diversity at the consensus-level among women in a given population (i.e., 80–95% of isolates among independent women were unique and differed by as few as 2 nucleotides or more) [11,13,14,23], and here we observe this across all HR-HPV types, which supports our hypothesis that the observed isolate switches may represent new HR-HPV isolate infections.

Across HR-HPV persistent infections, the cases that progressed to precancer/cancer during the study had significantly more conserved viral genomes, i.e., fewer within-host iSNV frequency changes, during persistent infection compared to the controls. This suggests that within-host viral genetic changes occurring during an infection tend to reduce the viability or fitness of the virus and thus reduce the risk of progression to precancer and cancer, consistent with the predominance of purifying selection and overall conservation of the HPV genome [9]. This observation agrees with a previous longitudinal study [16], which reported more iSNVs in the normal and low-grade diagnostic category compared to high-grade lesions. Genome conservation in the cases is consistent with the clonal expansion that is characteristic of cancer progression, which replaces a more homogeneous population of genomes with that of the expanding variant.

We observed that the E7 and E4 genes were particularly more conserved (i.e., fewer nonsynonymous single nucleotide changes) in persistent HR-HPV infections associated with progression to precancer/cancer compared to controls. This E7 association is consistent with our previous HPV16 study [11], and further expands this observation of E7 conservation and carcinogenesis to infections of all 12 HR-HPV types. Additionally, we have shown that nonsynonymous mutations in E7 are linked to more rapid viral clearance, suggesting that these variants are reducing the ability of the virus to persist and thus constrain the oncogenic potential of HR-HPV infections. A previous study has shown that nonsynonymous single nucleotide changes in HPV16 E7 can lead to lower E7 protein levels and defective cell transformation and growth [24]. Here, we have also taken a more granular approach by considering all single nucleotide changes at all VAFs and the genetic changes that occur during an infection between cases and controls, versus comparing only consensus E7 sequence differences. The HPV E4 protein is known to aid in virus synthesis and release by binding and disrupting keratin in the upper epithelial layer. Mutations to the HPV16 E4 protein have been previously reported and shown to disrupt its association with keratin [25]. Also, we and others have previously reported that the E4 protein in HPV16 is more constrained than E2 and shows disproportionately more synonymous mutations compared to E2 [9,11,26,27]. We make similar observations in this study, noting E4 conservation in persistent cases of all HR-HPV types and the Alpha-9 species, thereby suggesting that a conserved E4 protein is oncogenically more potent. Interestingly, in contrast to E7, the other two HPV oncoproteins, E5 and E6, showed the opposite pattern with more nonsynonymous mutations in cases, although only significant in E5. E5 is an accessory oncoprotein that downregulates the antiviral interferon response and disrupts MHC class I antigen processing and presentation [28]. A previous study has shown that certain nonsynonymous mutations in E5 can increase its thermodynamic stability and modulate its functional behavior [29], as well as lead to differential activation of the NF-kB signaling pathway that regulates cell proliferation and apoptosis. It’s possible that the cases with nonsynonymous mutations in E5 are more likely to have highly expressed NF-kB and progression.

Compared to previous longitudinal studies [13,21], our study provides greater resolution for understanding the changes accrued in the HPV genome during infection using ≥ 2 serial samples. For example, as previously reported with fewer time points [16], we also observe that certain iSNVs only appear at low frequencies (VAF < 10%) in some intervening serial samples but not in other serial time points. To investigate whether such low frequency iSNVs were sequencing artifacts, we manually inspected the sequence reads for a subset of these variants using IGV and confirmed they were good quality variants and consistent with being a true iSNV, as previously described [30]. Our study defined true iSNVs as those detected in at least two serial samples, following Mishra et al. 2024 [18], and we imposed stringent variant quality control criteria, including a minimum sequencing read depth of 100 for each iSNV site, to remove potential sequencing artifacts at low read depths [31]. Further, to test the robustness of our findings at different iSNV VAF thresholds, we also repeated the analyses using iSNVs with VAF ≥ 0.03 and ≤ 0.97 with consistent results. Lastly, to rule out the potential influence of low-level sublineage coinfections from the same HR-HPV type on iSNV VAF changes, we excluded all lineage/sublineage defining sites. Therefore, the HPV genome variation we observed is attributed to within-host changes acquired during infection.

Our results confirm that changes to the viral genome arise during infection in within-host HPV populations, i.e., present in only a minority of viral genomes. In the past, we have referred to such changes as within-host somatic variations [15] or as iSNVs [9,18]. Such variants may arise as an outcome of a constant interplay between elements of the host innate immune system (e.g., APOBEC3 proteins) and HPV, or as a simple consequence of random *de novo* mutation. While previous studies have observed relatively few consensus-level nucleotide substitutions in the HPV16 genome among serial samples of cases compared to controls [21,32], we observe the same phenomenon among cases at the within-host (sub-consensus) level. We note that we have not specifically probed the tri-nucleotide context of the iSNVs to verify if they exhibit APOBEC3 signatures [14,15,20,33], and we consider this an important area of future research.

Our study documents potential subclinical persistence (lack of detection) of HR-HPV type-specific infections, followed by re-appearance (detection) in 26 HR-HPV type infections that had an intervening negative within a persistent infection, i.e., an HPV-negative test in a string of persistent HPV-positive tests (i.e., skip infection). We verified that the viral genome isolates of the positive serial samples flanking the negative were the same exact sequence in 93% of the skip infections, suggesting that it was the exact same virus before and after the negative time point. Given the large amount of HPV isolate diversity observed in human populations [11,13,14], the likelihood of re-infection with the same exact isolate is minimal, supporting the explanation of subclinical persistence or lack of detection and subsequent re-appearance of the same infection. While the manifestation of subclinical persistence can be strongly influenced by certain factors, such as sample viral load, unifocal vs. multifocal infection, and sample collection technique (i.e., self-collection vs. collection by clinician), our consistent observation of identical genome isolates over many years of infection strongly suggests virological persistence below detection, attributable to host immune control, followed by infection re-appearance, rather than true clearance at the intervening time point [34].

We expect the effects of sequencing error on the iSNVs we observed to be minimal due to the very low single-base substitution error rate (~ 1 error per 10 kb sequenced [35]) that has been reported for our Ion Torrent sequencing platform. Additionally, our criterion of considering only those iSNVs consistently observed in two or more serial samples and considering the sites with sequencing depth ≥ 100 would further reduce the presence of sequencing errors, as previously reported [18,31]. However, our study has some limitations to note. Although our study is the largest to date to evaluate HPV genome variation across serial samples and the first study to evaluate all HR-HPV type genome variation, our sample size for each HR-HPV type and precancer/cancer cases were small. We observed significant associations between iSNV VAF changes and infection outcome when considering persistent infections across all the HR-HPV types, but the associations were not significant for each type, likely due to the small sample sizes. There is likely some variability among serial samples from cervical cell sampling (e.g., different viral loads) that we were unable to account for; not every cell of the cervix is the same and different “colonies” or populations of viruses may be represented in the samples. This could lead to misclassification of SNVs or co-infected within-type isolates from different viral populations in serial samples. However, we expect such sample heterogeneity to contribute equally to both the cases and controls, and we observed no correlation between sequence read depth and variant frequency. Therefore, this would not account for any of the case/control differences we observed.

## Conclusions

In summary, we comprehensively characterized HR-HPV genomic variation during persistent infection; determined that iSNV changes occur during infection and are linked to infection outcomes, possibly by reducing viral fitness or viability; and provided viral genomic evidence for persistent but intermittently undetected infections that were later re-detected. Importantly, HR-HPV type-level persistence can represent a heterogeneous collection of many viral isolates over time, which can appear as HPV type-specific persistence while including different infections. This underscores the limitations of classifying persistence solely at the HPV type level and highlights the need for higher-resolution genomic analyses incorporating isolate-level information, which may improve risk stratification in screening programs and inform the development and evaluation of therapeutic vaccine targets.

## Materials and methods

### Ethics approval and consent to participate

All the study participants provided written informed consent before participating in the study. The Guanacaste, Costa Rica Natural History Study was approved by the Institutional Review Board (IRB) of the National Cancer Institute (NCI), National Institutes of Health (NIH), and approved by the local IRB in Costa Rica.

### Study population

The Guanacaste, Costa Rica Natural History Study (NHS) has been previously described [36–38]. Briefly, the study included 10,049 randomly sampled adult women (18 + years) residing in the Guanacaste Province of Costa Rica who were recruited for screening and follow-up, from 1993 to 2002, to understand the natural history of HPV infection and cervical neoplasia. Any participant with an abnormal screening result was rescreened at an interval of 6–12 months, and women with normal or negative screening results were randomly assigned to either be rescreened every 6–12 months or for passive follow-up to be rescreened after five to seven years. Women with CIN2+ at enrollment were treated and not further followed. Cervical samples from the enrolled women were collected using broom devices and then placed into Digene specimen transport medium. MY09/M11 L1 primer PCR system (MY09/11 PCR) with AmpliTaq Gold polymerase was used for HPV genotyping of the cervical samples [37].

Here, we included 364 HR-HPV (HPV16, 18, 31, 33, 35, 39, 45, 51, 52, 56, 58, and/or 59) positive women from this cohort, with 2–10 serial samples per women (1,309 total serial samples) collected over 6 months to 8 years of follow-up. Cases were defined as women positive for HR-HPV and diagnosed with cervical intraepithelial neoplasia grade 2 or 3 (CIN2, n = 28; CIN3, n = 40), or cancer (n = 5). We included all women with CIN2+ and 2 or more HR-HPV positive serial samples; we included all available serial samples. We selected controls defined as women with type-specific HR-HPV positive persistence of 6 months to 8 years without progression to CIN2+ (≤ CIN1; n = 291) throughout the study period and included 2 (first and last available samples collected during follow-up) to 10 serial samples. We included 26 women that had a type-specific HR-HPV positive persistent infection with ≥ 1 intervening type-specific HR-HPV negative screening visits (flanked by HR-HPV positive visits; termed ‘skip infections’). Enrollment age of all selected women ranged from 18 to 89, with a median age of 34 years.

### HR-HPV whole-genome sequencing and variant calling

DNA was extracted from 1,309 HR-HPV positive serial samples. HPV16-positive serial samples were amplified using our previously described HPV16 single amplicon panel [39]. Serial samples positive for any of the remaining 11 HR-HPV types were amplified using an in-house custom assay, referred to as the **N**ational **C**ancer **I**nstitute **C**arcinogenic **H**PV **A**ll **N**ext **Ge**neration **S**equencing (NCI CHANGeS) assay. This assay uses a custom Ion AmpliSeq panel with 545 primer pairs, covering overlapping amplicons across the 12 HR-HPV types considered in this study. Primers were divided into two separate PCR reactions to avoid cross-reaction and primer dimerization. Amplicon lengths ranged from 69 bp to 324 bp, with a median length of 199 bp. Whole-genome sequencing was performed for all 12 HR-HPV types concurrently on the Ion PGM platform. Raw sequencing reads were quality-controlled and adaptor trimmed using the Torrent Suite Software and aligned to the HR-HPV reference genomes [5] using the Torrent Mapping Alignment Program (https://github.com/iontorrent/TS/tree/master/Analysis/TMAP). Only reads with a mapping quality of 4 and above were retained. Reference-genome-based variant calling was performed using the Torrent Variant Caller (TVC) (https://github.com/domibel/IonTorrent-VariantCaller).

VCF files were processed to decompose multi-allelic sites into bi-allelic records. Only single nucleotide variants were retained from the total pool of variants. SNPEff v3.6c [40] was used to annotate the variants for HPV gene or region and/or amino acid change.

A FASTA file with consensus viral genome sequences, including the samples that passed quality control and the nucleotide positions that were covered by at least 4 sequence reads, was created for each HR-HPV type along with sublineage references for each type. The FASTA file was used to build a phylogenetic tree with RAxML MPI [41], and lineages/sublineages were assigned based on the topology of the trees and proximity to the reference genomes for each HR-HPV type. Each sequence was assigned to one of the following lineages/sublineages for each HR-HPV type: HPV16 A1-4, B1-4, C1-4, D1-4; HPV18 A1-5, B1-B3, C; HPV31 A1-2, B1-2, C1-3; HPV33 A1-3, B, C; HPV35 A1-2; HPV39 A1-2, B; HPV45 A1-3, B1-2; HPV51 A1-4, B1-2; HPV52 A1-2, B1-2, C1-2, D; HPV56 A1-2, B; HPV58 A1-3, B1-2, C, D1-D2; HPV59 A1-3, B [5,12,42–44].

Of the 364 women included in our study (576 HR-HPV type-specific infections, including type co-infections), we excluded 87 HR-HPV type-specific infections that failed HPV sequencing and another 93 that had data from only a single serial time point (180 total type-specific infections excluded). Therefore, we included 396 infections with ≥ 2 serial samples from 351 women in our analyses (n = 1,228 serial samples) (Fig 1).

### HR-HPV infection categories by viral genome or isolate persistence

Based on changes to the viral genome sequence across serial samples for a given woman, we classified each of the 396 HR-HPV type-specific infections into one of the following categories. Nucleotide changes observed in the low-complexity sequence region between the E5 and L2 genes were excluded from this analysis.

- *Isolate Persistence* – Infection persistence was first identified at the HR-HPV type level and then using the viral genome sequence data for that type, we evaluated viral persistence among the serial samples at the isolate level (i.e., viral consensus genome sequence persistence). An infection was classified as persistent at the isolate level if there were no changes (i.e., exact same genome sequence) or one single-nucleotide change was observed in the viral genome sequence in all the serial samples of that woman. A viral genome sequence was considered a unique isolate if it differed from the other serial samples’ sequences by two or more nucleotide positions, and these infections were not considered persistent at the isolate level. The 27 persistent infections with a type-specific intervening HPV-negative sample were considered skip rather than persistent infections (see below).
- *Isolate Switch/Coinfection* – HR-HPV type persistent infections that had more than one viral isolate sequence detected among serial samples were classified as isolate switches, and/or as having a coinfection of more than one isolate of the same HR-HPV type.
- *Skip infection/intervening HPV-negative* – A HR-HPV type-specific persistent infection that was HPV-negative at ≥ 1 intervening samples between HPV-positive samples in the flanking earlier and later screening visits was classified as a skip infection. The skip infection serial sample sequences were compared at the consensus level and subsequently manually reviewed at a finer resolution by two independent reviewers, who inspected nucleotide positions and sequence reads using the Integrative Genomics Viewer (IGV) [45]. Every nucleotide position was manually reviewed across the entire genome for each serial sample to determine if the same exact viral genome was present at all time points and specifically in the flanking HPV-positive samples. One of the 27 identified skip infections was excluded from analysis due to insufficient viral genome coverage in the flanking samples for evaluation.

### HR-HPV infection categories by outcome

Each persistent HR-HPV type-specific infection was also classified based on the worst diagnosis and/or viral clearance (i.e., undetectable infection) during the study period. Each woman’s visit history report was manually reviewed to determine if she progressed to a high-grade lesion (CIN2 or higher) during the study, and if not, if she cleared her infection or had a persistent infection without viral clearance. For those that did not progress, we estimated the time between the visit date that first tested positive for the HR-HPV type and the date she tested HPV-negative. Using this information, the following categories were used to define the infection outcomes:

- *Control/Clearance*: The HR-HPV type-specific infection was cleared or was no longer detected (HPV-negative) without progression to CIN2+.
- *Control/No clearance*: The HR-HPV type-specific infection persisted and never cleared or progressed to CIN2+ during the study period.
- *Case:* HR-HPV type-specific infection that progressed to a high-grade lesion (CIN2+).
- *Others*: HR-HPV type-specific infection that cleared, however the woman progressed to CIN2+ due to a co-infection with a different type or due to a subsequent HR-HPV infection.

### Estimating VAF changes among serial samples for each iSNV

We evaluated all single nucleotide variants (SNVs) with sequencing depth ≥ 100 that were detected in at least 2 serial samples. To remove potential sequencing errors that may occur at a very low frequency, variants with VAF < 0.005 were set to 0 and those with VAF > 0.995 were set to 1. Variants occurring at sublineage-defining positions for a given HR-HPV type (positions that differ between the sublineage reference sequences for that specific type) were excluded. From this set of variants, we only considered the iSNV sites (within-host variable sites) with VAF ≥ 0.005 and ≤ 0.995 in at least one time point.

iSNV VAF changes were calculated for all persistent HR-HPV infections by evaluating changes across all positive serial samples for each persistent infection. First, the VAF for each detected iSNV was calculated for each serial visit. If an iSNV was not detected at a given visit, its VAF was set to 0. Changes in iSNV VAF over time (hereafter, denoted as ΔiSNV_VAF_) were then estimated as the slope of a linear regression line (VAF as a function of number of days since first visit). A positive slope suggests increasing VAF during the infection period, a negative slope suggests decreasing VAF.

To assess the significance of the observed VAF change, a null distribution was generated for ΔiSNV_VAF_ by randomly selecting a subset of iSNVs, shuffling their VAF, and calculating ΔiSNV_VAF_ for all (shuffled and not shuffled) iSNVs. The procedure was repeated multiple times to generate a null distribution of VAF change. Separate null distributions were generated for HPV16 and the other HPV types to account for assay differences. To account for variability in VAF change due to the number of visits, the null distributions were stratified based on whether the iSNV was observed in 2 visits or > 2 visits. Each observed ΔiSNV_VAF_ was assigned a percentile rank based on its null distribution by type (HPV16/non-HPV16) and number of visits (n = 2 or n > 2 visits). A 10th/90th percentile cutoff was applied to identify iSNVs that significantly increased (percentile rank ≥ 90) or decreased (percentile rank ≤ 10) in VAF. Each persistent HR-HPV infection was then classified as either *VAF-change* or *No-VAF-change* infection. Infections with at least one iSNV exhibiting a significant ΔiSNV_VAF_ were classified as VAF-change infections (S4 Fig); all others were classified as No-VAF-change.

### Evaluating nonsynonymous mutations by HPV gene

All single-nucleotide variants (both iSNVs and SNVs) in HR-HPV persistent infections were annotated for synonymous and nonsynonymous amino acid changes across HPV genes (E6, E7, E1, E2, E4, E5, L1, and L2 ORFs) using annotations from PAVE [46]. The annotated variants were subsequently screened for evidence of sublineage coinfection. To identify variant sites in a serial sample consistent with low-level sublineage coinfection within the same HR-HPV type, single nucleotide variants with VAF < 0.002 were set to 0 and VAF > 0.98 were set to 1. Only variable iSNV sites (considered as those having a VAF between 0 and 1 in at least one serial sample) with sequencing depth ≥ 100, observed in at least 2 serial samples and that were also sublineage defining positions were further considered. These iSNV sublineage defining sites were then clustered based on VAF of the minor allele (VAF < 0.5). We expect sublineage defining haplotype variants (i.e., linked variants) will have similar VAF values, and therefore, cluster together. To this end, we clustered all iSNV sublineage defining sites whose minor allele VAFs were within 0.05 of each other. Next, only those identified sites that were from potential sublineage coinfections with iSNV clusters of > 10 iSNVs were excluded. The remaining single nucleotide changes (iSNVs and SNVs) within these infections were included for this analysis. Only infections with ≥ 3 nonsynonymous mutations were considered for analysis, and the distribution of nonsynonymous mutations was compared between the Control/Clearance and Case (CIN2+) groups.

### Statistical analysis

Differences in the HPV type distribution by age group, infection outcome, and number of serial samples were evaluated by Fisher’s exact tests. A Wilcoxon rank-sum (Mann-Whitney) test was used to compare age as a continuous variable for the women with skip infections compared to those without. Fisher’s exact tests were used to estimate the odds ratio (OR), 95% confidence intervals (95% CI) and significance (P value) of the associations between iSNV changes and disease risk, using the controls (≤CIN1) as the referent group. Associations with disease risk were performed using logistic regression models without adjustment and with adjustment for age at enrollment, number of sexual partners, and HR-HPV type coinfection (yes/no). Significance level was considered at 5% and statistical tests were two-sided. Statistical analyses were performed with R version 4.2.3.

## Supporting information

S1 File Nonsynonymous variants by HPV type and gene. List of the nonsynonymous single nucleotide variants with VAF ≥ 0.005, sequencing depth ≥100 and appearing in at least 2 serial samples. These were the variants included in the analysis illustrated in Fig 5. Columns descriptions: HPV_TYPE, the HR-HPV type; POS, nucleotide position; REF, reference nucleotide; ALT, variant nucleotide; AA mutation, amino acid change and position; Gene, HPV gene that includes the variant; Sublineage-defining site (Yes/No), if this position is considered a sublineage-defining site for the specified HR-HPV type; No. cases with variant, number of CIN2+ cases with the variant; No. controls with variant, number of controls with the variant; Total cases by HPV type, total number of CIN2+ cases with the specified HR-HPV type; Total controls by HPV type, total number of control with the specified HR-HPV type.
(XLSX)

S1 Table Characteristics of the 351 women in the Guanacaste, Costa Rica Natural History Study by HR-HPV type. (PDF)

S2 Table Summary of the frequency of viral persistence at the isolate-level by the duration of the infection (0–2 years or >2 years) for each HR-HPV type across all type-persistent infections. (PDF)

S3 Table Nonsynonymous single nucleotide changes associated with infection outcome by viral gene for Alpha-9 HPV types. (PDF)

S4 Table Summary of each skip infection with one or more intervening HPV-negative tests by HR-HPV type. (PDF)

S1 Fig Distribution of nucleotide differences among serial sample viral isolates (n = 1,193). The percentage of isolates across all infections that differ by 0, 1, 2, 3, 4, 5 and > 5 nucleotide positions from the consensus sequence of each infection is shown.
(PDF)

S2 Fig Distribution of isolate diversity among the women for each HR-HPV type. The percentage of consensus sequences with 0–1 and >1 nucleotide differences is shown for each HR-HPV type (n = 369 infections from 331 women). Nucleotide differences were calculated by exhaustive pairwise comparisons of enrollment sample (i.e., one sample per woman) viral isolate sequences between all the women positive for the HR-HPV type.
(PDF)

S3 Fig VAF change by depth. (A) The correlation between the standard deviation of flow total depth (FDP) and standard deviation of VAF, and (B) correlation between VAF and FDP of each serial iSNV observation are shown. The linear regression/correlation line is shown in red. iSNV, intrahost single nucleotide variant; VAF, variant allele fraction.
(PDF)

S4 Fig Approach to classify infections into VAF-change/No-VAF-change status. Each HR-HPV type-specific infection was classified based on whether it had at least one iSNV with significant increase/decrease in variant allele fraction (VAF) (VAF-change) or no iSNVs with significant VAF change (No-VAF-change).
(PDF)

S5 Fig Distribution of infection outcomes by HR-HPV type. The % of the total for the distribution of each HR-HPV type is shown considering 364 HR-HPV persistent infections. Control:Clearance includes infections that cleared/controlled and the woman never progressed to a cervical intraepithelial neoplasia grade 2 or higher (CIN2+); Control:No clearance includes infections that never cleared/controlled during the course of the study and the woman never progressed to a CIN2+; Cases includes infections that progressed to CIN2+; Others are the infections that cleared, but the woman progressed to CIN2 due to the presence of a different HR-HPV infection.
(PDF)

S6 Fig Distribution of HR-HPV control infections by viral clearance time. The number of years between the date the HR-HPV type was first detected (HR-HPV positive) and the date it was no longer detected (HR-HPV negative, date of clearance) for the 153 infections classified as control:clearance.
(PDF)

S7 Fig Distribution of nonsynonymous single nucleotide variants (both iSNVs and fixed SNVs) by gene and infection outcome for the Alpha-9 types (A) and A1/A2 sublineages across all HR-HPV types (B). Only infections with ≥3 nonsynonymous changes were considered. P values were corrected using FDR correction; only significant p values are shown. The L2 gene panel shows the number of infections for each outcome category. Case includes cervical intraepithelial neoplasia grade 2 or higher (CIN2+). E6, early gene 6; E7, early gene 7; E1, early gene 1; E2, early gene 2; E4, early gene 4; E5, early gene 5; L2, late gene 2; L1, late gene 1; * (p < 0.05), ** (p < 0.01).
(PDF)

S8 Fig Age of the 26 women with skip infections compared to those without skip infections (n = 369 HR-HPV infections). Wilcoxon two-sided test p value = 0.29.
(PDF)
