# Supplementary material for: Longitudinal analysis of high-risk HPV infections reveals within-host viral genome changes over time
Source: PLoS Pathog. 2026 Jul 15;22(7):e1014362. doi: 10.1371/journal.ppat.1014362 (PMC13372122; doi:10.1371/journal.ppat.1014362)
Supplement: S1 Table — (PDF) [file ppat.1014362.s002.pdf]

Characteristics	HPV Type 16 N (%)	18 N (%)	31 N (%)	33 N (%)	35 N (%)	39 N (%)	45 N (%)	51 N (%)	52 N (%)	56 N (%)	58 N (%)	59 N (%)	Total (col %)	P
**Enrollment Age**														
18-29	39 (25.8)	15 (9.9)	28 (18.5)	6 (4)	2 (1.3)	5 (3.3)	5 (3.3)	10 (6.6)	10 (6.6)	12 (7.9)	17 (11.3)	2 (1.3)	151 (38.0)	
30-39	15 (16.5)	6 (6.6)	13 (14.3)	5 (5.5)	5 (5.5)	6 (6.6)	8 (8.8)	3 (3.3)	5 (5.5)	10 (11)	14 (15.4)	1 (1.1)	91 (23.0)	
40-49	16 (32.7)	3 (6.1)	3 (6.1)	2 (4.1)	2 (4.1)	2 (4.1)	1 (2)	0 (0)	9 (18.4)	5 (10.2)	5 (10.2)	1 (2)	49 (12.4)	0.19
50-59	7 (18.4)	3 (7.9)	3 (7.9)	4 (10.5)	2 (5.3)	2 (5.3)	0 (0)	4 (10.5)	3 (7.9)	5 (13.2)	4 (10.5)	1 (2.6)	38 (9.6)	
60+	17 (25.4)	4 (6)	7 (10.4)	7 (10.4)	5 (7.5)	1 (1.5)	2 (3)	5 (7.5)	7 (10.4)	6 (9)	5 (7.5)	1 (1.5)	67 (17)	
**Histology**														
≤ CIN1	62 (19.7)	25 (7.9)	45 (14.3)	20 (6.3)	14 (4.4)	15 (4.8)	12 (3.8)	19 (6)	29 (9.2)	33 (10.5)	36 (11.4)	5 (1.6)	315 (79.5)	
CIN2	8 (24.2)	4 (12.1)	3 (9.1)	2 (6.1)	1 (3)	1 (3)	4 (12.1)	2 (6.1)	0 (0)	2 (6.1)	6 (18.2)	0 (0)	33 (8.3)	0.10
CIN3	22 (50)	1 (2.3)	6 (13.6)	2 (4.5)	1 (2.3)	0 (0)	0 (0)	1 (2.3)	4 (9.1)	3 (6.8)	3 (6.8)	1 (2.3)	44 (11.1)	
Cancer	2 (50)	1 (25)	0 (0)	0 (0)	0 (0)	0 (0)	0 (0)	0 (0)	1 (25)	0 (0)	0 (0)	0 (0)	4 (1)	
**No. of serial samples**														
2	41 (23.6)	14 (8)	31 (17.8)	11 (6.3)	3 (1.7)	7 (4)	9 (5.2)	14 (8)	14 (8)	10 (5.7)	17 (9.8)	3 (1.7)	174 (43.9)	
3	20 (23.5)	4 (4.7)	9 (10.6)	6 (7.1)	5 (5.9)	1 (1.2)	3 (3.5)	4 (4.7)	10 (11.8)	7 (8.2)	14 (16.5)	2 (2.4)	85 (21.5)	
4	9 (15.5)	4 (6.9)	7 (12.1)	2 (3.4)	5 (8.6)	6 (10.3)	0 (0)	2 (3.4)	6 (10.3)	11 (19)	5 (8.6)	1 (1.7)	58 (14.6)	0.06
5+	24 (30.4)	9 (11.4)	7 (8.9)	5 (6.3)	3 (3.8)	2 (2.5)	4 (5.1)	2 (2.5)	4 (5.1)	10 (12.7)	9 (11.4)	0 (0)	79 (19.9)	
Total	94 (23.7)	31 (7.8)	54 (13.6)	24 (6.1)	16 (4)	16 (4)	16 (4)	22 (5.6)	34 (8.6)	38 (9.6)	45 (11.4)	6 (1.5)	396 (100)	

Infections from all the 351 women included in our study are included in this table, a total of 396 HR-HPV type-specific infections. Women positive for >1 HR-HPV types were counted more than once. P value (P) was calculated using Fisher's test with 100,000 Monte-Carlo iterations to allow convergence.
CIN1 is cervical intraepithelial neoplasia (CIN) grade 1; CIN2, CIN grade 2; CIN3, CIN grade 3; Cancer includes adenocarcinoma and squamous cell carcinoma.
