# Supplementary material for: Longitudinal analysis of high-risk HPV infections reveals within-host viral genome changes over time
Source: PLoS Pathog. 2026 Jul 15;22(7):e1014362. doi: 10.1371/journal.ppat.1014362 (PMC13372122; doi:10.1371/journal.ppat.1014362)
Supplement: S2 Table — (PDF) [file ppat.1014362.s003.pdf]

HPV type	Total No. infections	Duration of infection*	Isolate persistence No. (%)	Isolate switch/coinfections No. (%)
HPV16	87	0-2 years<br>> 2 years	7 (70%)<br>38 (49.3%)	3 (30%)<br>39 (50.7%)
HPV18	29	0-2 years<br>> 2 years	3 (100%)<br>21 (80.8%)	0 (0%)<br>5 (19.2%)
HPV31	50	0-2 years<br>> 2 years	8 (88.9%)<br>36 (87.8%)	1 (11.1%)<br>5 (12.2%)
HPV33	22	0-2 years<br>> 2 years	1 (20%)<br>9 (52.9%)	4 (80%)<br>8 (47.1%)
HPV35	15	0-2 years<br>> 2 years	3 (100%)<br>10 (83.3%)	0 (0%)<br>2 (16.7%)
HPV39	13	0-2 years<br>> 2 years	2 (100%)<br>8 (72.7%)	0 (0%)<br>3 (27.3%)
HPV45	15	0-2 years<br>> 2 years	1 (50%)<br>12 (92.3%)	1 (50%)<br>1 (7.7%)
HPV51	20	0-2 years<br>> 2 years	3 (100%)<br>13 (76.5%)	0 (0%)<br>4 (23.5%)
HPV52	33	0-2 years<br>> 2 years	5 (83.3%)<br>27 (100%)	1 (16.7%)<br>0 (0%)
HPV56	34	0-2 years<br>> 2 years	7 (100%)<br>26 (96.3%)	0 (0%)<br>1 (3.7%)
HPV58	45	0-2 years<br>> 2 years	10 (100%)<br>32 (91.4%)	0 (0%)<br>3 (8.6%)
HPV59	6	0-2 years<br>> 2 years	1 (50%)<br>3 (75%)	1 (50%)<br>1 (25%)

*Duration of infection is defined as the number of years between the date the HR-HPV type was first detected (HR-HPV positive) and the date it was no longer detected (HR-HPV negative, date of clearance). Isolate persistence is defined as the same consensus HR-HPV genome sequence observed in all serial samples (0 or 1 nucleotide change), and an isolate switch is defined as a different consensus HR-HPV genome sequence with ≥ 2 nucleotide changes observed across serial samples.
