# Supplementary material for: Longitudinal analysis of high-risk HPV infections reveals within-host viral genome changes over time
Source: PLoS Pathog. 2026 Jul 15;22(7):e1014362. doi: 10.1371/journal.ppat.1014362 (PMC13372122; doi:10.1371/journal.ppat.1014362)
Supplement: S3 Table — (PDF) [file ppat.1014362.s004.pdf]

HPV Type	Gene	Infection outcome*	No Nonsynonymous changes (%)	≥ 1 Nonsynonymous changes (%)	OR (95% CI)	P value#
Alpha-9	**E5**	Clearance (0-2 yrs)	11 (84.6)	2 (15.4)	Ref	
		CIN2+	16 (32.7)	33 (67.3)	10.88 (2.03,112.59)	**0.008**
	**E7**	Clearance (0-2 yrs)	4 (30.8)	9 (69.2)	Ref	
		CIN2+	3 (16.7)	15 (83.3)	0.2 (0.04,0.86)	0.069
	**E6**	Clearance (0-2 yrs)	9 (69.2)	4 (30.8)	Ref	
		CIN2+	16 (32.7)	33 (67.3)	4.52 (1.06,23.26)	0.069
	**E4**	Clearance (0-2 yrs)	5 (38.5)	8 (61.5)	Ref	
		CIN2+	32 (65.3)	17 (34.7)	0.34 (0.07,1.39)	0.226
	**E1**	Clearance (0-2 yrs)	6 (46.2)	7 (53.8)	Ref	
		CIN2+	20 (40.8)	29 (59.2)	1.24 (0.3,5.05)	1
	**E2**	Clearance (0-2 yrs)	1 (7.7)	12 (92.3)	Ref	
		CIN2+	4 (8.2)	45 (91.8)	0.94 (0.02,10.72)	1
	**L1**	Clearance (0-2 yrs)	2 (15.4)	11 (84.6)	Ref	
		CIN2+	7 (14.3)	42 (85.7)	1.09 (0.1,6.93)	1
	**L1**	Clearance (0-2 yrs)	1 (7.7)	12 (92.3)	Ref	
		CIN2+	6 (12.2)	43 (87.8)	0.6 (0.01,5.77)	1

Only infections with ≥ 3 nonsynonymous mutations were considered.
'No Nonsynonymous changes is the number of infections with no nonsynonymous iSNVs/SNVs, '≥ 1 Nonsynonymous changes is the number of infections with at least 1 nonsynonymous iSNV/SNV.
Odds ratios (OR), 95% confidence intervals (95% CI) and P values were calculated using a two-sided Fisher's exact test; to enable calculations for Fisher exact test, the counts were incremented by 0.6 if any count in a 2x2 contingency table was 0.
Ref: referent group.
CIN2+ cases include cervical intraepithelial neoplasia grade 2 or higher.
FDR correction was applied to the P values; significant p values (<0.05) are shown in bold.
*Coinfected cases with sequencing data were excluded.
