# Supplementary material for: Longitudinal analysis of high-risk HPV infections reveals within-host viral genome changes over time
Source: PLoS Pathog. 2026 Jul 15;22(7):e1014362. doi: 10.1371/journal.ppat.1014362 (PMC13372122; doi:10.1371/journal.ppat.1014362)
Supplement: S4 Table — (PDF) [file ppat.1014362.s005.pdf]

HPV type	HPV sublineage	Same flanking isolate	No. intervening negative samples	Years of intervening negative	Enrollment age
HPV33	A1	Yes	1	2	32
HPV56	A1	Yes	1	1.7	33
HPV33	A1	Yes	3	5.2	59
HPV51	A1	Yes	2	4.4	59
HPV39	A1	Yes	3	4.1	29
HPV16	A1	Yes	2	6.5	24
HPV16	A1	Yes	1	2	71
HPV16	A1	Yes	1	1.1	32
HPV31	A1	Yes	2	1.8	74
HPV45	B2	Yes	1	2.2	35
HPV31	C3	Yes	1	1.5	23
HPV18	A3	Yes	2	3.2	41
HPV16	A2	No	1	2.2	29
HPV18	B3	Yes	1	0.9	56
HPV56	A1	Yes	1	1.5	38
HPV31	A1	Yes	1	1.4	20
HPV39	A1	Yes	1	2.8	27
HPV52	D	Yes	2	2.8	62
HPV39	A1	Yes	1	2.1	52
HPV56[#]	B	Yes	1	1.2	47

HPV56#	B	Yes	1	1.7	47
HPV51	A1	Yes	1	2	71
HPV16	A1	Yes	1	1.5	24
HPV16	D2	Yes	3	4.2	44
HPV31	C	Yes	1	2.2	22
HPV35*	A1	Yes	2	1.9	67
HPV35*	A2	No (sublineage switch)	1	2.1	67

#,* Two independent skip infections were observed in the same HR-HPV infection. Years of intervening negative is the number of years between positive year after skip and positive year before skip.
