# Supplementary figures and images for: Longitudinal analysis of high-risk HPV infections reveals within-host viral genome changes over time
Source: PLoS Pathog. 2026 Jul 15;22(7):e1014362. doi: 10.1371/journal.ppat.1014362 (PMC13372122; doi:10.1371/journal.ppat.1014362)

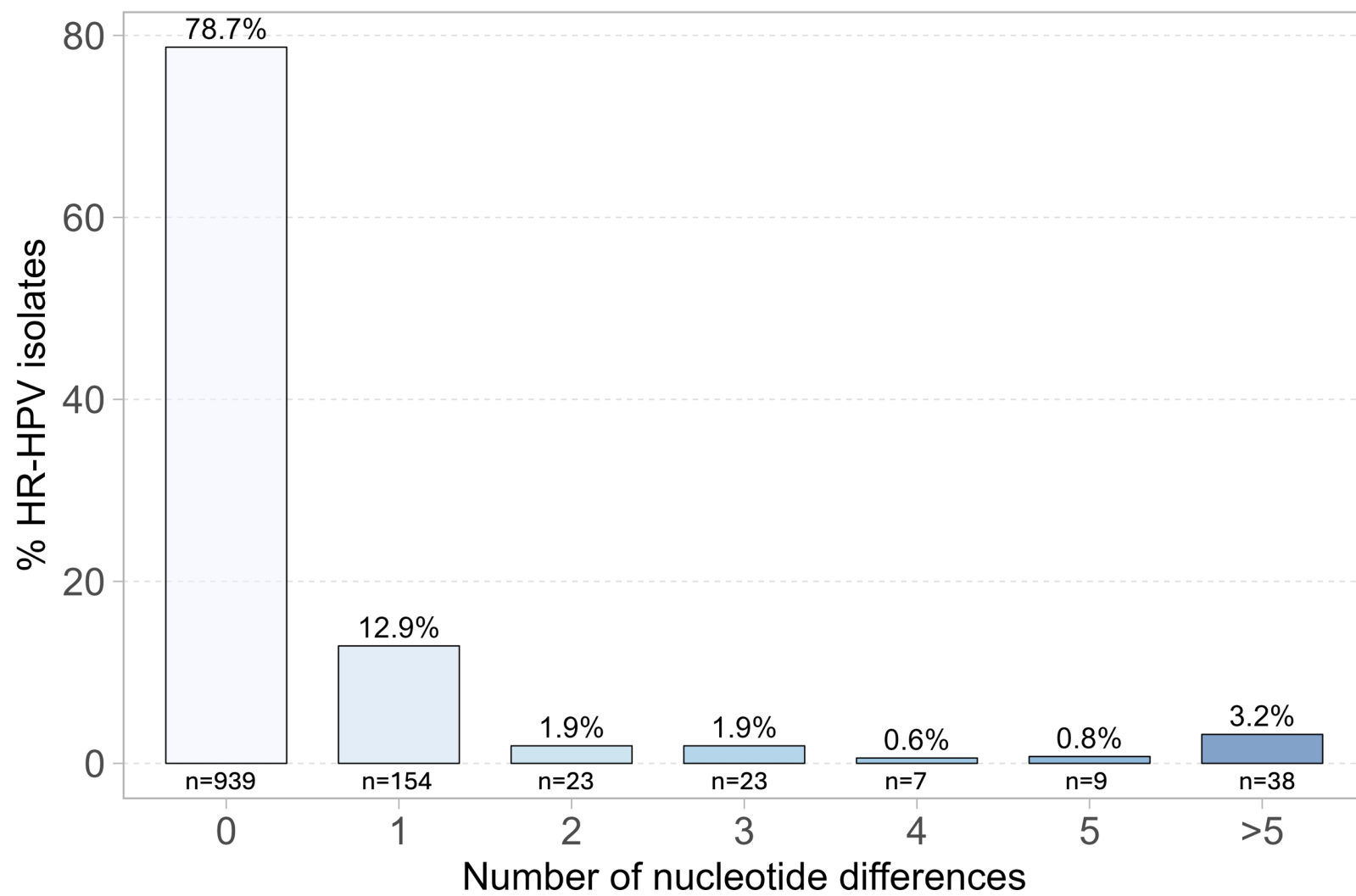

Supplement: S1 Fig — The percentage of isolates across all infections that differ by 0, 1, 2, 3, 4, 5 and > 5 nucleotide positions from the consensus sequence of each infection is shown. (PDF) [file ppat.1014362.s006.pdf]

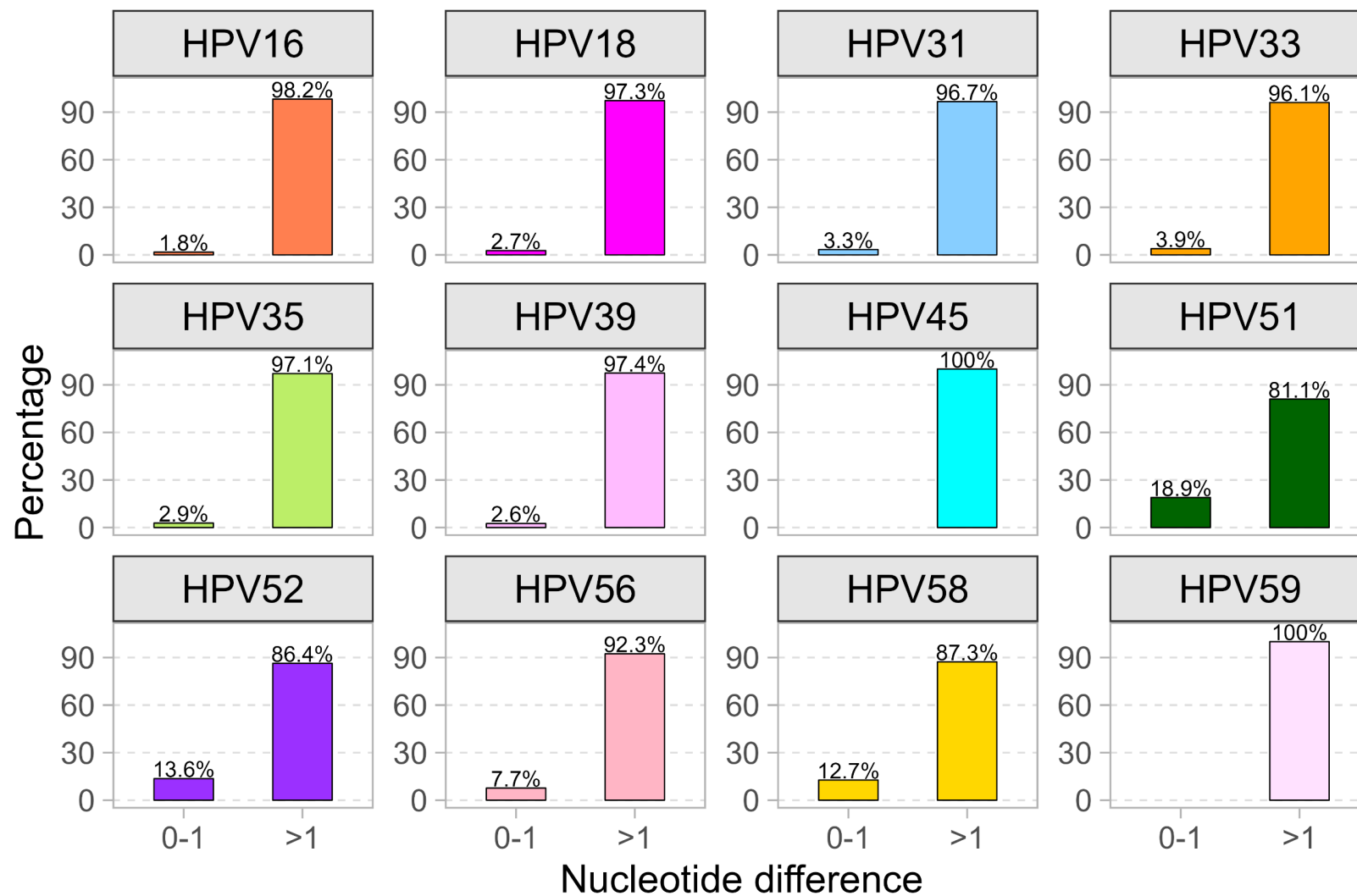

Supplement: S2 Fig — The percentage of consensus sequences with 0–1 and >1 nucleotide differences is shown for each HR-HPV type (n = 369 infections from 331 women). Nucleotide differences were calculated by exhaustive pairwise comparisons of enrollment sample (i.e., one sample per woman) viral isolate sequences between all the women positive for the HR-HPV type. (PDF) [file ppat.1014362.s007.pdf]

**A**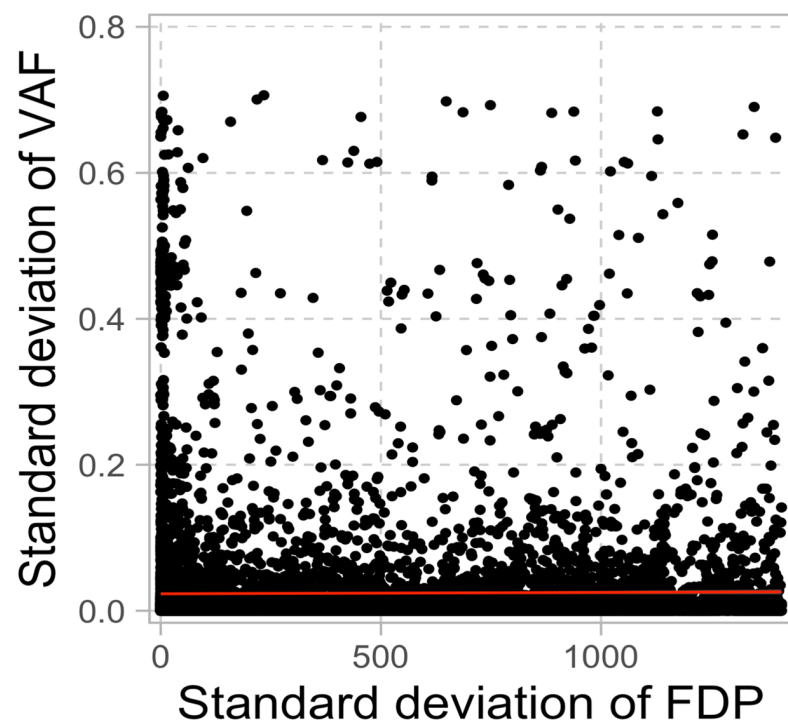**B**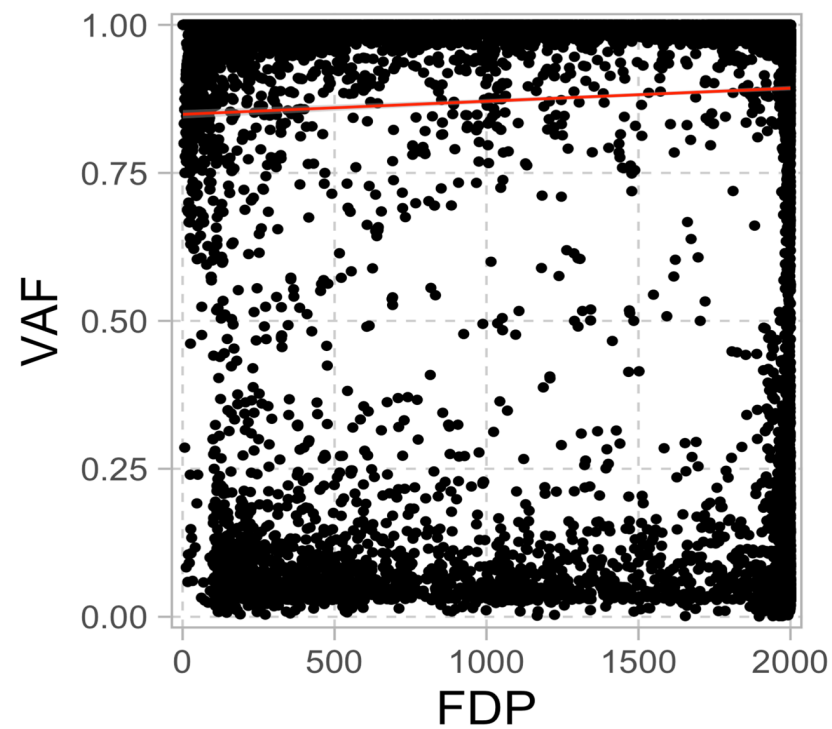

Supplement: S3 Fig — (A) The correlation between the standard deviation of flow total depth (FDP) and standard deviation of VAF, and (B) correlation between VAF and FDP of each serial iSNV observation are shown. The linear regression/correlation line is shown in red. iSNV, intrahost single nucleotide variant; VAF, variant allele fraction. (PDF) [file ppat.1014362.s008.pdf]

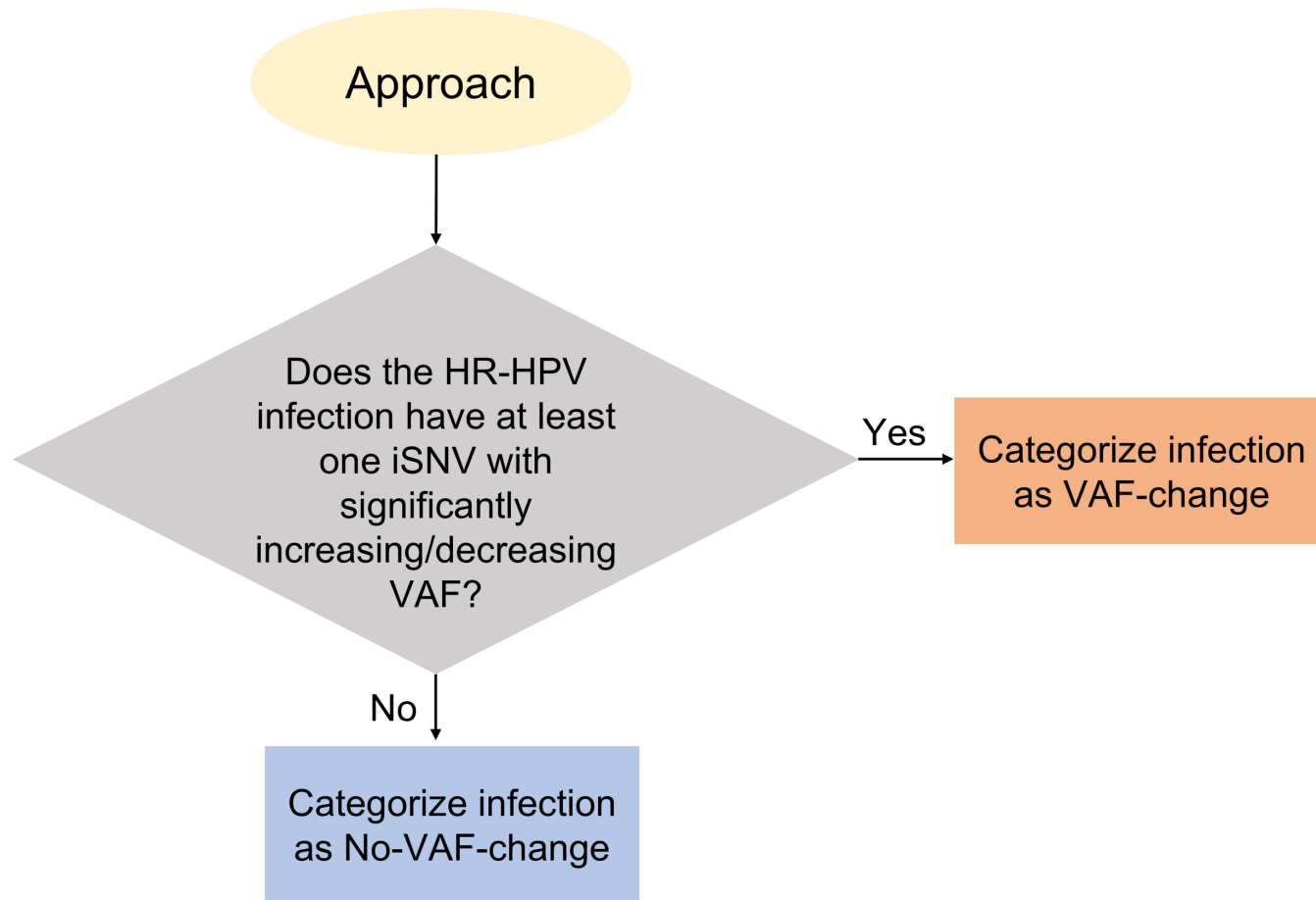

Supplement: S4 Fig — Each HR-HPV type-specific infection was classified based on whether it had at least one iSNV with significant increase/decrease in variant allele fraction (VAF) (VAF-change) or no iSNVs with significant VAF change (No-VAF-change). (PDF) [file ppat.1014362.s009.pdf]

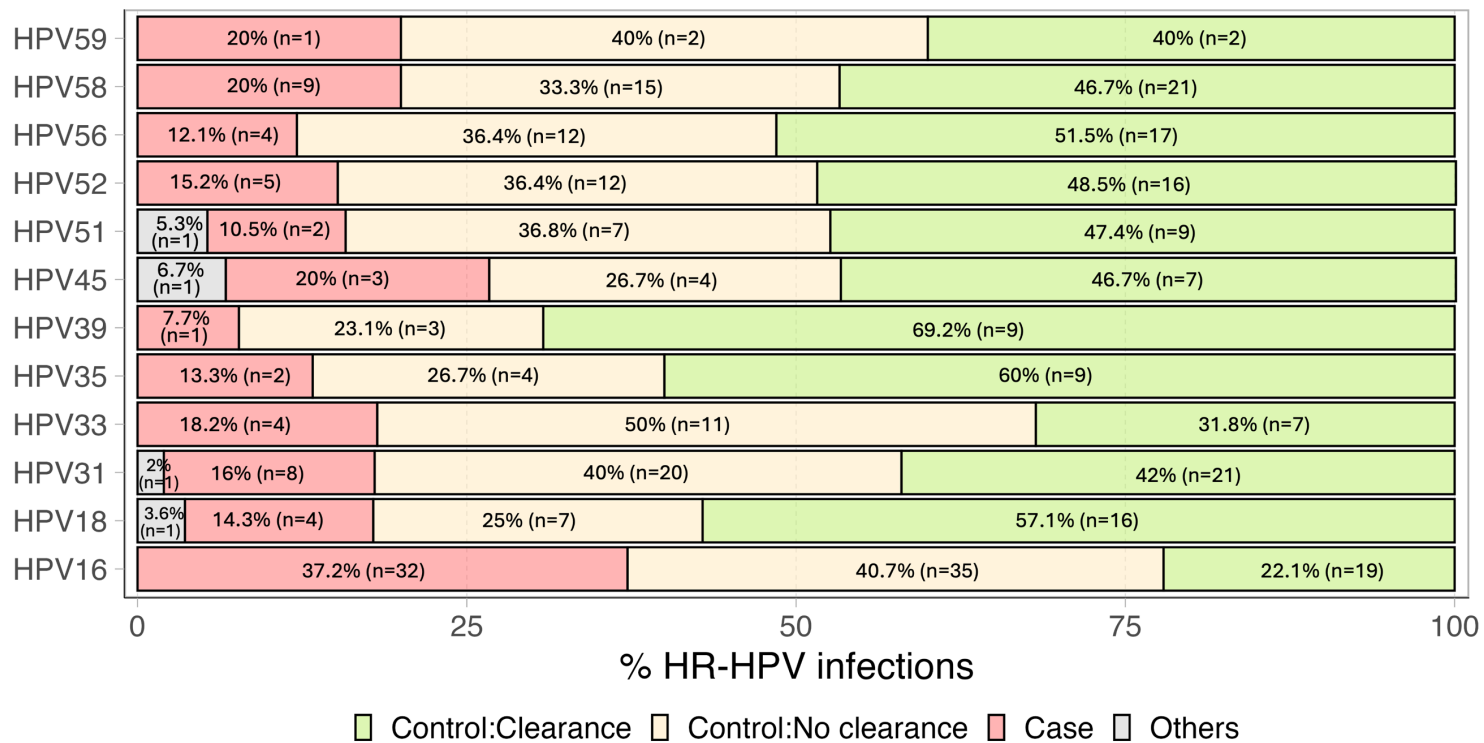

Supplement: S5 Fig — The % of the total for the distribution of each HR-HPV type is shown considering 364 HR-HPV persistent infections. Control:Clearance includes infections that cleared/controlled and the woman never progressed to a cervical intraepithelial neoplasia grade 2 or higher (CIN2+); Control:No clearance includes infections that never cleared/controlled during the course of the study and the woman never progressed to a CIN2+; Cases includes infections that progressed to CIN2+; Others are the infections that cleared, but the woman progressed to CIN2 due to the presence of a different HR-HPV infection. (PDF) [file ppat.1014362.s010.pdf]

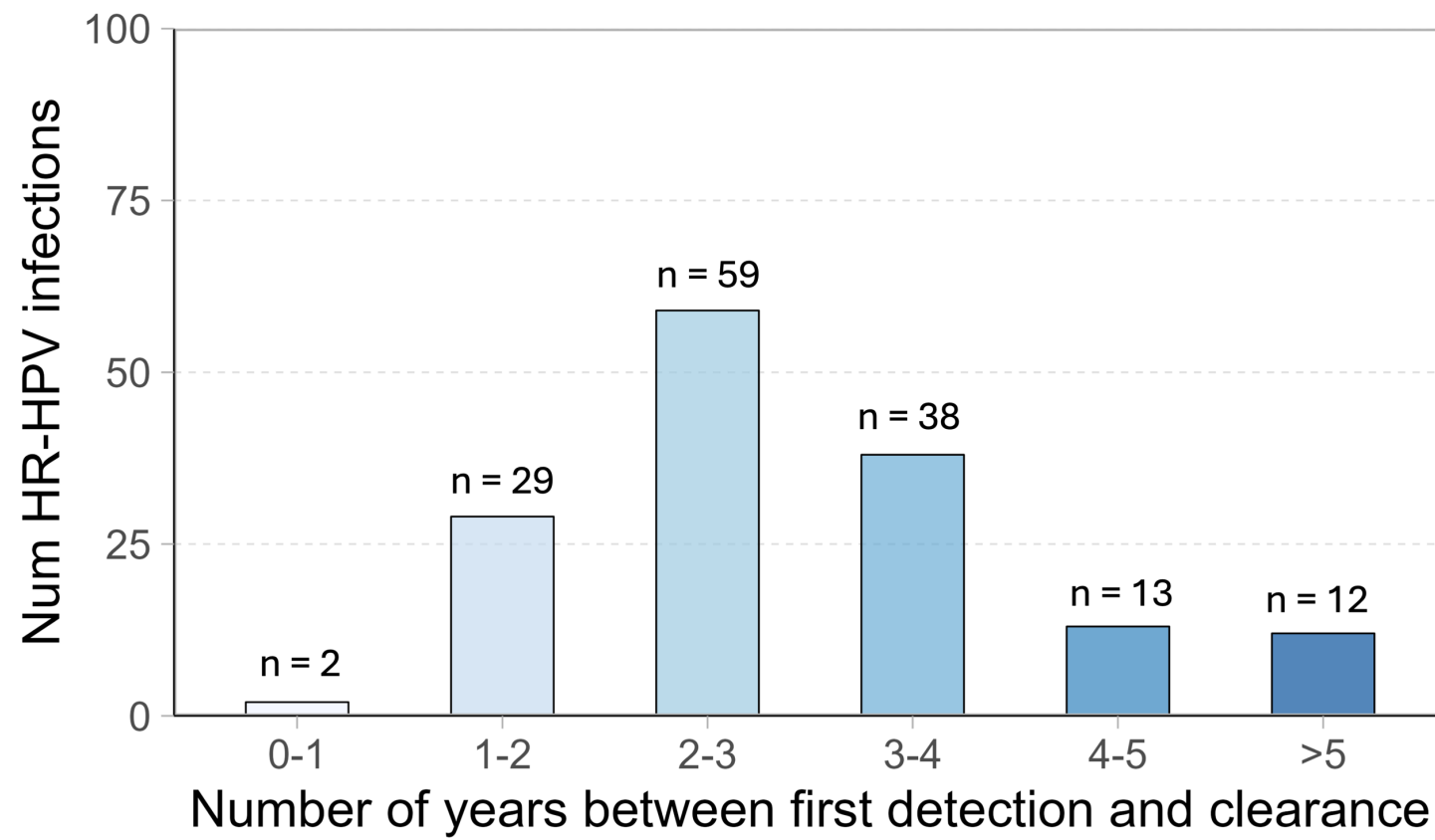

Supplement: S6 Fig — The number of years between the date the HR-HPV type was first detected (HR-HPV positive) and the date it was no longer detected (HR-HPV negative, date of clearance) for the 153 infections classified as control:clearance. (PDF) [file ppat.1014362.s011.pdf]

A

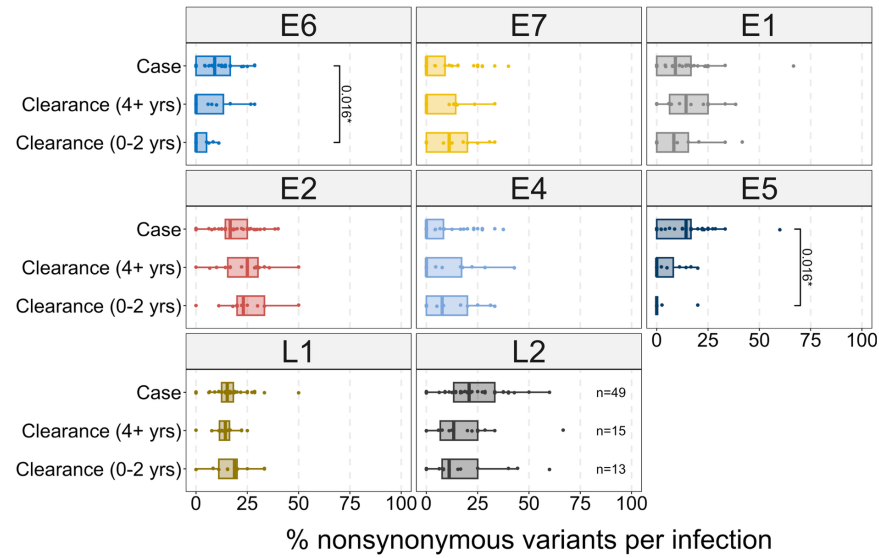

B

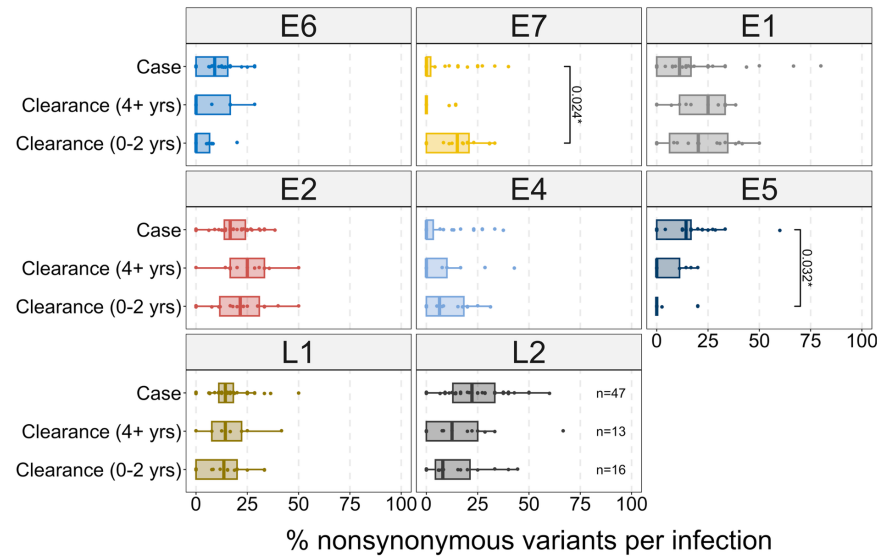

Supplement: S7 Fig — Only infections with ≥3 nonsynonymous changes were considered. P values were corrected using FDR correction; only significant p values are shown. The L2 gene panel shows the number of infections for each outcome category. Case includes cervical intraepithelial neoplasia grade 2 or higher (CIN2+). E6, early gene 6; E7, early gene 7; E1, early gene 1; E2, early gene 2; E4, early gene 4; E5, early gene 5; L2, late gene 2; L1, late gene 1; * (p < 0.05), ** (p < 0.01). (PDF) [file ppat.1014362.s012.pdf]

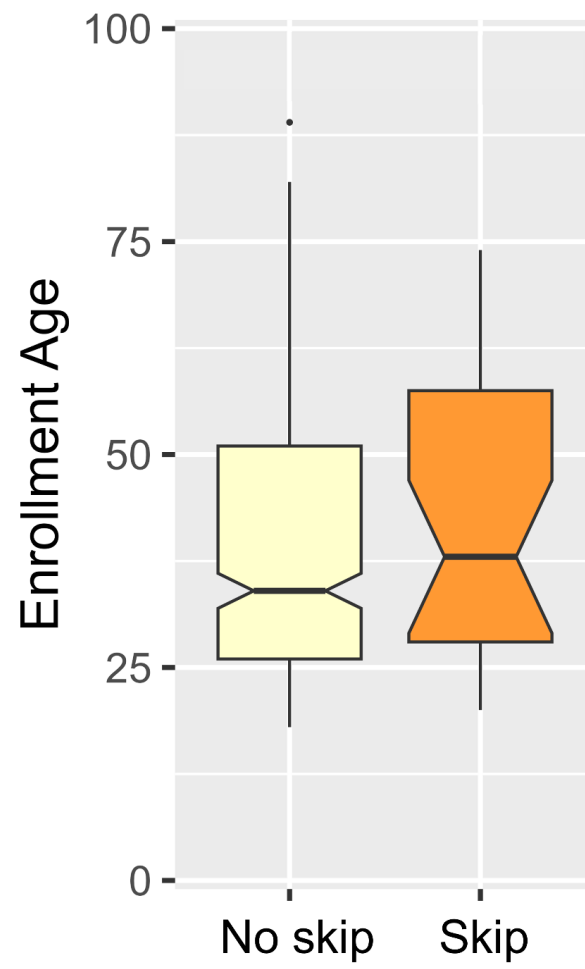

Supplement: S8 Fig — Wilcoxon two-sided test p value = 0.29. (PDF) [file ppat.1014362.s013.pdf]
